# Differential expression and function of CAIX and CAXII in breast cancer: A comparison between tumorgraft models and cells

**DOI:** 10.1371/journal.pone.0199476

**Published:** 2018-07-02

**Authors:** Zhijuan Chen, Lingbao Ai, Mam Y. Mboge, Chingkuang Tu, Robert McKenna, Kevin D. Brown, Coy D. Heldermon, Susan C. Frost

**Affiliations:** 1 The Department of Biochemistry and Molecular Biology, University of Florida, Gainesville, FL, United States of America; 2 The Department of Medicine, University of Florida, Gainesville, FL, United States of America; University of South Alabama Mitchell Cancer Institute, UNITED STATES

## Abstract

Carbonic anhydrase IX (CAIX) and XII (CAXII) are transmembrane proteins that are associated with cancer progression. We have previously described the catalytic properties of CAIX in MDA-MB-231 breast cancer cells, a line of cells that were derived from a patient with triple negative breast cancer. We chose this line because CAIX expression in breast cancer is a marker of hypoxia and a prognosticator for reduced survival. However, CAXII expression is associated with better survival statistics than those patients with low CAXII expression. Yet CAIX and CAXII have similar catalytic activities. Here we compare the potential roles of CAIX and CAXII in the context of TNBC and estrogen receptor (ER)-positive breast cancer. In tumor graft models, we show that CAIX and CAXII exhibit distinct expression patterns and non-overlapping. We find the same pattern across a panel of TNBC and luminal breast cancer cell lines. This affords an opportunity to compare directly CAIX and CAXII function. Our data suggest that CAIX expression is associated with growth potentiation in the tumor graft model and in a TNBC line using knockdown strategies and blocking activity with an impermeant sulfonamide inhibitor, N-3500. CAXII was not associated with growth potentiation. The catalytic activities of both CAIX and CAXII were sensitive to inhibition by N-3500 and activated at low pH. However, pH titration of activity in membrane ghosts revealed significant differences in the catalytic efficiency and pKa values. These features provide evidence that CAIX is a more efficient enzyme than CAXII at low pH and that CAIX shifts the equilibrium between CO_2_ and bicarbonate in favor of CO_2_ production by consuming protons. This suggests that in the acidic microenvironment of tumors, CAIX plays a role in stabilizing pH at a value that favors cancer cell survival.

## Introduction

There is a strong correlation between lactic acid production and metastatic incidence [1]. Yet, glycolysis-deficient cancer cells still generate “acidic” tumors when injected into nude mice [2, 3]. With lactic acid reduced by 30% in these tumors, metabolic profiling revealed that CO_2_ may be an additional source of acidity [4]. These data imply a contribution by carbonic anhydrase (CA) which catalyzes the reversible hydration of CO_2_ to bicarbonate and a proton. In humans, there are 13 active members in this group that differ in their kinetic and inhibitory properties, cell and tissue distribution, and function [5, 6]. The focus of this study is on two membrane-bound CAs (CAIX and CAXII).

We, and others, have shown that CAIX is a homodimeric transmembrane glycoprotein oriented with the catalytic domains facing the extracellular milieu [7–9]. The domain structure of mature CAIX contains a proteoglycan-like domain (PG-like), a catalytic domain (CA), a transmembrane domain (TM), and a cytoplasmic tail (CT) [7]. CAIX is normally expressed in gut epithelial tissue [10, 11] but is upregulated in several forms of cancer, including breast cancer. Indeed, CAIX is a marker for hypoxic regions of breast tumors [12] and elevated expression is associated with poor prognosis [13–15] and high-grade, ER-negative breast tumors [13]. The only tumor type in which CAIX expression is an indicator of more favorable patient outcome is in renal clear cell carcinoma [16] although this view is controversial [17].

CAXII has a secondary structure and orientation similar to that of CAIX, but lacks the PG-like domain. In breast cancer cells, CAXII expression is regulated by estrogen [18, 19]. All of the clinical breast cancer studies to date have shown a correlation between CAXII expression and better survival statistics for patients [18–21]. Indeed, these later studies show a strong association between luminal cancers and CAXII expression. CAXII is also a biomarker of favorable prognosis in lung [22] and brain [23] tumors, but is associated with poor prognosis in colorectal cancer [24]. Despite these obvious differences, CAIX and CAXII have similar activities suggesting that their native environments may influence their activities and/or biochemical function.

Because the catalytic domains of CAIX and CAXII are oriented to the extracellular milieu, both could serve as therapeutic targets. However, CAIX has received more attention because of its expression in aggressive cancers, including triple negative breast cancer. Here we present a comparative study of CAIX and CAXII to understand better the roles of CAIX and CAXII in breast cancer. We show that CAIX and CAXII exhibit differential expression in tumor graft models of the three major subtypes of breast cancer and that expression of CAIX and CAXII are mutually exclusive. Studies in breast cancer cells confirm this observation. In addition, our data show that only CAIX expression is associated with cell growth and migration. Finally, we present data that confirms that the activity profile of CAIX over CAXII is favored at pH values associated with acidic tumors.

## Materials and methods

### Animal ethics statement

After cell/tissue implantation to initiate tumor growth, Carprofen (5mg/kg) was injected subcutaneously every 24 h for 3 days to ameliorate pain and suffering when necessary. For tumor excision, animals were provided isoflurane (2% inhalant with O_2_) as an anesthetic. At the end of tumor excision, animals were euthanized by CO_2_ inhalation from a compressed gas cylinder. The animals were placed in a cage/chamber and then CO_2_ was slowly added to the chamber until the animal ceases to breathe. The animal underwent cervical dislocation to assure death.

### Cell culture

Triple negative breast cancer (TNBC) cell lines: UFH-001 cells were established in our lab [25], MDA-MB-231-LM2 cells were gift from Dr. Dietmar Seimann (University of Florida), HBL-100 and BT549 cells were gifts from Dr. Brian Law (University of Florida), and SUM159 cells were purchased from Asterand Bioscience. UFH-001, MDA-MB-231-LM2, HBL100, and BT549 cells were plated and maintained in Dulbecco’s Modified Eagle’s medium (DMEM) supplemented with 10% fetal bovine serum (FBS) (Sigma Aldrich #F2442). SUM159 cells were cultivated in Ham’s F-12 medium (Corning Cellgro, #10-080-CM) supplemented with 10% FBS, 5μg/mL insulin (Gibco, A1138211), 100ng/mL dexamethasone (Sigma Aldrich, #D4902) and 10mM HEPES (Sigma Aldrich, #H-4034).

ER positive (ER^+^) cell lines: T47D, MCF7 and SKBR cells were gifts from Dr. Brian Law (University of Florida), and SUM52 cells were from Asterand Bioscience. T47D and SKBR cells were plated and maintained in McCoys 5A medium (Fisher #16-600-108), supplemented with 10% FBS and 10μg/mL insulin. MCF-7 cells were plated and maintained in DMEM supplemented with 10% FBS and 0.01μM estrogen (Sigma Aldrich, #E8875). SUM 52 cells were cultivated in Ham’s F-12 medium supplemented with 10% FBS, 5μg/mL insulin, 100ng/mL dexamethasone and 10mM HEPES. Cancer-associated fibroblasts (CAFs) were provided by Dr. Song Han (University of Florida). CAFs were plated and maintained in DMEM/Ham’s F12 medium supplemented with 10% FBS. All cell lines were maintained at 37°C in humidified air with 5% CO2.

### Co-culture of CAFs and breast cancer cell lines (HBrCs)

To avoid direct cell-cell contact, CAFs were first plated in 6-well plates at a density of 3x10^5^ cells/well. Within 1 hour of CAFs plating, UFH-001 or T47D cells were plated on 6-well Trans-well inserts (0.4um) at a density of 3x10^4^ cell/insert and 6x10^4^ cell/insert respectively. CAFs and breast cancer cells were co-cultured at 37°C in 5% CO_2_ for 5 days.

### Knockdown of CAIX and CAXII by shRNA lentiviral particles

Knockdown of CAIX and CAXII expression in UFH-001 and T47D cells was performed by transfection of short hairpin RNA (shRNA) lentiviral particles (Thermo scientific) against CAIX and CAXII. In brief, breast cancer cells were seeded at 5x10^4^ cells/well in 24-well plates and grown for 24 hours. Then, cells were infected with lentivirus in serum-free medium for 6 hours. The shRNA target sequences for CAIX and CAXII are shown in S1. The cells were further incubated with normal growth medium for 24 h. GFP expression was monitored to confirm the efficiency of transduction. Stable cells were established by puromycin (2 μg/mL) selection (Sigma Aldrich #P-7255). CAIX and CAXII knockdown was confirmed by western blotting.

### CAIX deletion by CRISPR/Cas9

LentiCRISPR v2 was a gift from Dr. Feng Zhang (Addgene plasmid #52961) and was used to knockout CAIX in UFH-001 cells. Guide RNA sequences within the first coding exon were identified downstream of the CAIX translational start site using an online design tool (crispr.cos.uni-heidelberg.de) and three non-overlapping gRNAs were chosen within this region of the *CA9* gene (S2 Table). Complementary double-stranded oligonucleotides were cloned into BsmBI digested plentiCRISPR v2, recombinant clones identified by restriction analysis and confirmed by automated Sanger sequencing. Lentivirus were formed from these CAIX sgRNA plasmids and empty lentiCRISPR v2 plasmid as previously outlined [26]. UFH-001 cells were transduced and selected with puromycin selection for 3 weeks. Stably transduced cells were harvested and CAIX depletion was confirmed by western blotting.

### Tumor graft models (PDX)

Frozen and cryo-preserved PDX tissues, originally isolated from human breast tumor tissue, were provided by Dr. Alana Welm (Huntsman Cancer Institute, University of Utah). These samples were provided after multiple passages of tissue across mice and do not require an IRB. The description by which the tissues were collected and serially transferred in mice, along with their molecular characterization, has been previously published [27]. Subsequently, we have generated our own PDXs, on site. For the TNBC PDX model (HCI-001), a single fragment of cryopreserved tumor tissue (~8 mm^3^) was implanted into the 4^th^ mammary fat pad of 10-12-week-old female NOD-SCID mice (Jackson Laboratory, ME, USA). For ER-positive PDX (HCI-011), estrogen pellets were implanted into the 4^th^ mammary fat pad of female NOD-SCID mice to provide estrogen supplementation just prior to tissue transplantation. When the tumor volume reached 500 mm^3^, the mice were sacrificed and dissected tumor was used for immunohistochemistry. All procedures were conducted in accordance with the National Institutes of Health regulations and approved by the University of Florida Institutional Animal Care and Use Committee (IACUC # 201603567). Frozen PDX samples were homogenized in RIPA buffer containing protease inhibitor (Sigma # P8340) using an Omni homogenizer. Protein concentrations were determined by the Markwell modification [28] of the Lowry method [29] and used to determine protein loading for SDS-PAGE analysis.

### Xenograft models

All procedures were conducted in accordance with the National Institutes of Health regulations and approved by the University of Florida Institutional Animal Care and Use Committee (IACUC protocol # 201603567). For mouse xenografts, a total of 3×10^5^ UFH cells (EV controls, CAIX-KO #3 and CAIX-KO #4) was suspended in culture medium was injected into the fourth left mammary fat pad of NOD/SCID mice (Jackson Laboratory, ME, USA), aged around 10–12 weeks old. Tumor sizes were measured with a digital caliper (Fisher Scientific, MA, USA), and calculated volume was calculated using the formula: 0.5 x L x W^2^ (L- length, W-width). The Xenogen IVIS Spectrum system (Caliper Lifesciences, MA, USA) was used for *in vivo* imaging to track tumor growth and metastasis.

### Western blot analysis

Western blot analysis was performed as previously described [25]. In brief, cultured cells were washed with ice-cold PBS and lysed in RIPA buffer at 4°C containing a cocktail of protease inhibitors (Sigma-Aldrich, P8340). Lysates were clarified by centrifugation. The supernatants were collected and total protein concentration was determined [28]. After SDS-PAGE, proteins were transferred onto nitrocellulose membranes for western blot analysis. Membranes were incubated with specific antibodies: CAIX (M75 monoclonal, gift from Dr. Egbert Oosterwijk), CAII (Novus, #NB600-919), CAXII (R&D, # AF2190), estrogen receptor (Santa Cruz, # sc-8002), E-cadherin (BD biosciences, #610181), GAPDH (Cell Signaling, #5174), and actin (Abcam, #ab8226) followed by incubation with horseradish peroxidase–conjugated secondary antibodies (Sigma-Aldrich). The secondary antibodies were detected by enhanced chemiluminescence (GE Healthcare Biosciences, #RPN2106).

### Immunohistochemistry

Samples of PDX tissue were fixed in 4% paraformaldehyde and embedded in paraffin. Immunohistochemistry was performed on sectioned paraffin-embedded tumor tissue. After de-paraffination and rehydration, slides were blocked with a mouse IgG then were incubated with primary antibodies to CAIX, CAXII, ER or Ki67 (Dako #M7240) followed by the application of peroxidase-conjugated secondary antibody. Detection of CAIX, CAXII, ER and Ki67 was achieved by incubating slides in 3’ 3’ diaminobenzidine for 1 minute at RT. Slides were counterstained with hematoxylin for 30 s and mounted with Cytoseal XYL.

### Mass spectrometry and ^18^O exchange analysis

CA catalyzes the reversible hydration of CO_2_ to bicarbonate and a proton. We used an Extrel EXM-200 mass spectrophotometer to measure the rate of exchange of ^18^O from CO_2_ and bicarbonate to water at chemical equilibrium [30–33]. Cells were collected from culture plates using cell release buffer (Gibco, #13151–014). Cells were extensively washed with bicarbonate-free DMEM buffered with 25 mM Hepes (pH 7.4) and counted. Then, cells (5 ×10^5^ cells/mL) were added to a reaction vessel containing 2 mL of buffered, bicarbonate-free DMEM at 16°C in which was dissolved ^18^O-enriched ^13^CO_2_/H^13^CO_3_^-^ at 25 mM total ^13^CO_2_ species. The atom fraction of ^18^O in extracellular ^13^CO_2_ was detected by the membrane inlet mass spectrometry. Loss of ^18^O in CO_2_ species is a specific reflection of CA activity. The impermeant sulfonamide CA inhibitor, N-3500, was used to demonstrate specificity of the exofacial CA activity. The properties of this inhibitor and a detailed description of this technique, including the calculations of kinetic values, has been published previously [34, 35]. Enzyme concentration was estimated using the tight binding sulfonamide inhibitor, ethoxzolamide.

### Cell proliferation assays

UFH-001 parental cells and CAIX-KO UFH-001 cells were plated at a density of 1500 cells/well into 96-well plates. T47D parental cells and CAXII-KO T47D cells were plated at a density of 5000 cells/well into 96-well plates. UFH-001 and T47D cells were treated with N-3500. Cell growth was determined by using a standard tetrazolium bromide (MTT) assay on sequential days after plating.

### Cell migration and invasion assays

Empty vector and CAIX knockdown UFH-001 cells, empty vector and CAXII knockdown T47D cells were exposed to serum-free medium for 24 h. Cells were released from plates with enzyme-free cell dissociation buffer. They were then plated in serum-free medium (specific for cell type) at a density of 50,000 cells/300 μL/insert in 24-well cell migration and invasion plates (Cell Biolabs, CBA-100-C-5). The cells were allowed to migrate (24 h) or invade (48 h) across the insert towards the bottom well, which had medium containing 10% FBS. The assay was terminated after the appropriate time point. Cells were fixed, stained, and photographed for image analysis. All cell lines were maintained at 37°C in humidified air with 5% CO_2_ for the duration of the experiment.

### Statistical evaluation

Student’s t test was used to compare the properties of CA activities, cell growth, migration, and invasion within and across cell lines. All *p* values were based on two-tailed analysis and *p* < 0.05 was considered as statistically significant. Statistical analysis was performed using Prism software.

## Results

### CA expression in breast cancer as a survival predictor

**Fig 1:**
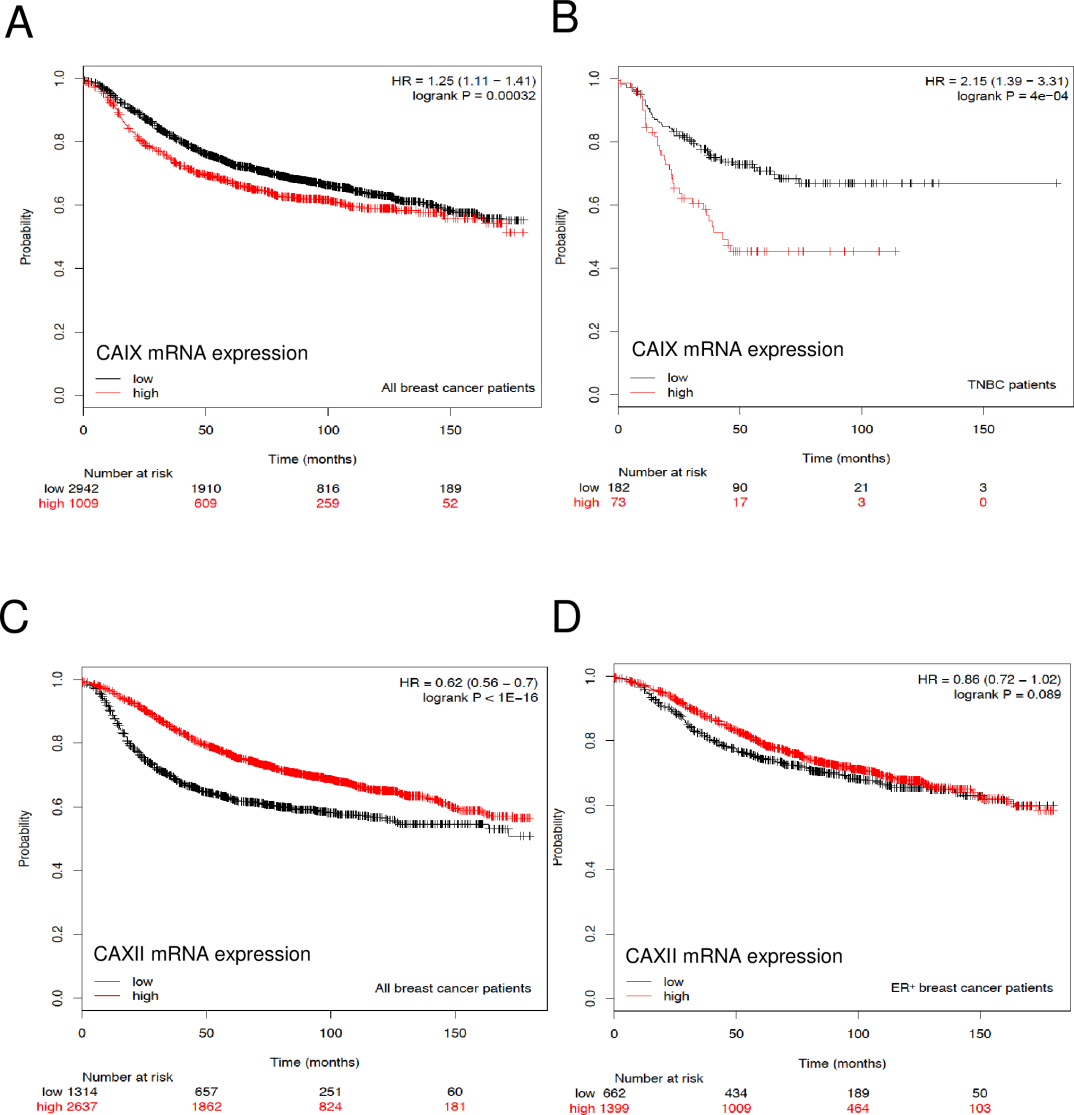
Kaplan-Meier plots in breast cancer patients. *Panel A*. mRNA from all breast cancer patients (unrestricted analysis) was probed for the *CA9* gene expression (CAIX-mRNA) using Affimetrix ID 205199_at. *Panel B*. mRNA expression in triple negative breast cancer patients was probed for CAIX mRNA using Affimetrix ID, 205199. *Panel C*. mRNA from all breast cancer patients (unrestricted analysis) was probed for the *CA12* gene expression (CAXII mRNA) using Affimetrix ID, 215867_at. *Panel D*. mRNA from all ER-positive breast cancer patients was probed for the CAXII mRNA using Affimetrix ID, 215867_at.

To examine the association of CA gene expression on patient survival, we used the publically available Kaplan-Meier database (kmplot.com/analysis/) which is comprised of information downloaded from GEO (Affymetrix microarrays only, EGA, and TCGA). The web tool that is used to query these databases was developed by Lanczky et al. [36]. Breast cancer patients that have high expression of CAIX mRNA have lower disease-free survival (RFS) rates than those with low expression (*p* = 0.00032) (Fig 1A). The subpopulation that has the greatest risk are those associated with the TNBC phenotype (*p* = 4e-04) (Fig 1B). In contrast, breast cancer patients that show high expression of CAXII mRNA show higher RFS rates relative to those with low expression (*p* = 8.3e-13) (Fig 1C). This holds true for the estrogen receptor positive patients (*p* = 0.0056) (Fig 1D), although surprisingly not as impressive as for all breast cancer patients. While these data suggest a fairly clear association between CAIX and CAXII mRNA with specific subtypes of breast cancer, the survival data vary significantly when other subtypes are evaluated for CAIX or CAXII mRNA expression (S1 Fig and S2 Fig). Because TNBC patients (Fig 1B) are usually the basal subtype, CA9 mRNA expression in the basal breast cancer patients predicts reduced survival (S1A Fig). However, for luminal A or luminal B subtype (S1B–S1D Fig), elevated CAIX mRNA expression is actually a more positive prognosticator for RFS. CAIX mRNA expression in patients with HER2 expression is also associated with improved survival, but did not reach statistical significance. Surprisingly, CAXII mRNA expression is associated with reduced RFS in patients with the basal phenotype (S2A Fig). While the same trend is observed for HER2 positive patients (S2B Fig), the numbers of patients are low and the difference in survival fails to reach significance. The survival curves of luminal A and B patients expressing CAXII mRNA (S2C and S2D Fig) resemble the survival curves for ER-positive patients (Fig 1D) and predict extended survival. The question that remains is whether changes in the mRNA pool leads to changes in protein expression, which ultimately might change the physiology of the tumor.

### CAIX and CAXII expression in the PDX model

**Fig 2:**
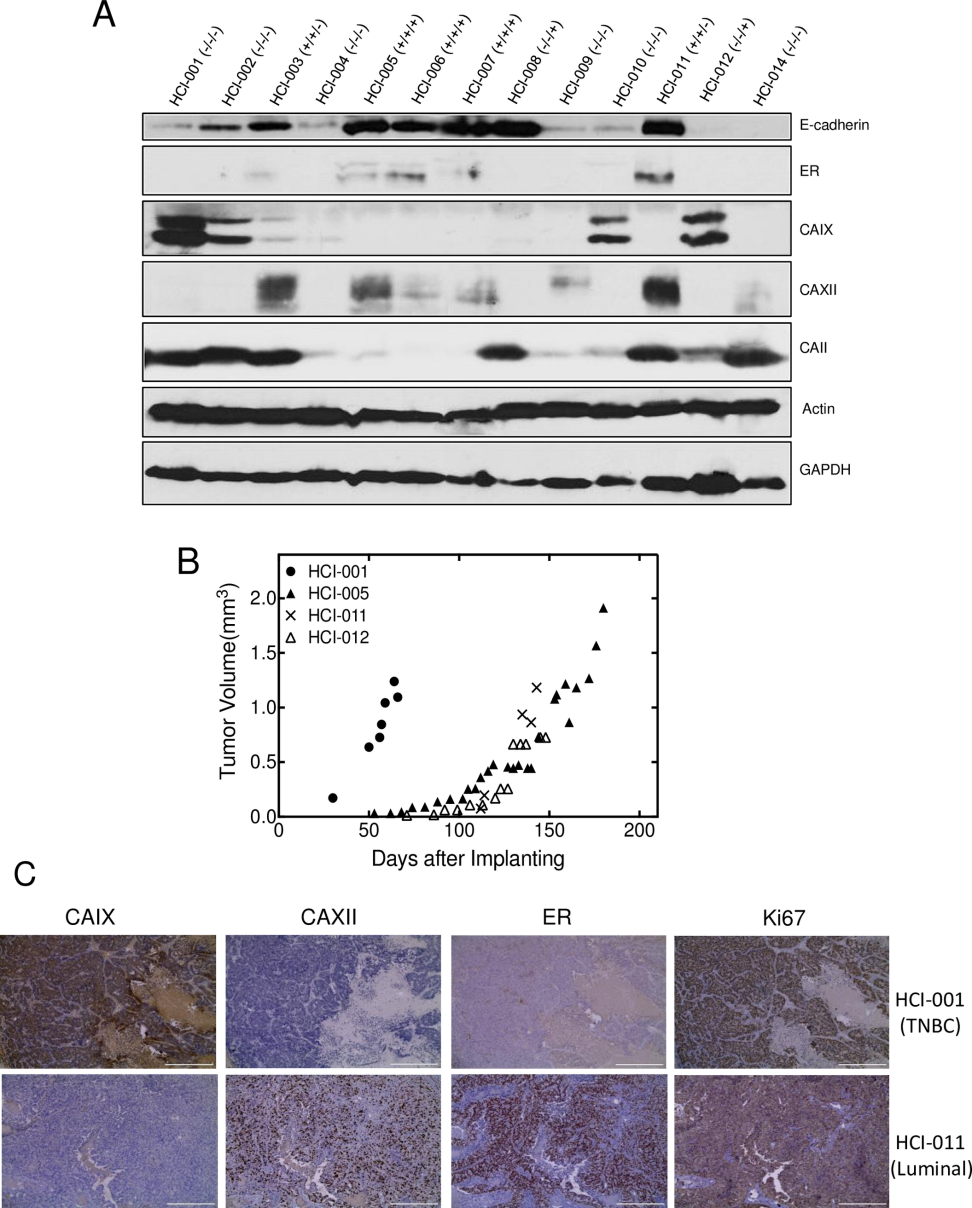
Differential expression of CAIX and CAXII in patient-derived tumor grafts. Panel A. Frozen tissue samples from PDX tumors were homogenized in RIPA buffer containing protease inhibitor. Western blots were probed with antibodies for CAIX, CAXII, CAII, ER, E-cadherin, actin, and GAPDH. Panel B. Tumor growth rates of orthotopically implanted, cryo-preserved tumor tissue was evaluated in NOD/SCID mice. Panel C. Immunohistochemistry was utilized to evaluate expression of CAIX, CAXII, ER, and Ki67 in TNBC (HCl-001) and ER/PR-positive (HCl-011) tumors from Panel B. Magnification: primary objective magnification, 10x.

To further explore CA protein expression in breast cancer patients, we utilized the tumor grafting model (PDX) developed in the Welm lab [27]. We evaluated 13 samples of frozen tissue which were derived from patients diagnosed with ER-positive, HER2-positive or triple negative breast cancers. Only two of the tissue samples (HCl-003 and HCl-011) were derived from ER/PR positive patients (Fig 2A). Both samples expressed CAXII. These samples had little or no CAIX expression. Three additional patient-derived samples also expressed CAXII (HCl-005, HCl-006, and HCl-007). These were isolated from ER/PR- and HER2-positive patients. These tissue samples also show CAXII expression but not CAIX. Of the six triple negative samples (HCl-001, HCl-002, HCl-004, HCl-009, HCl-010 and HCl-014), three showed strong expression of CAIX (HCl-001, HCl-002 and HCl-010) (Fig 2A). No CAXII was detected in these samples. Two other samples were derived from HER2-positive patients (HCl-008 and HCl-012). Only one of these showed strong expression of CAIX and in neither sample was there CAXII expression. While these data sets are small, they suggest that there is no co-expression of CAIX with CAXII in breast cancer tumors. If this holds true, then the better predictor for patient outcome will come from protein expression arrays (vs RNAseq or microarrays). The well-studied cytosolic CA, CAII, is expressed in all tissue samples except HCI-007, although the level of expression appears to vary across samples. This may be related to contamination of tumor tissue with red blood cells, which contain substantive amounts of CAII.

We also used cryopreserved tissue provided by the Welm lab to generate our own PDX mice. The growth potential of tumors derived across breast cancer phenotypes is shown in Fig 2B. It is clear that the tissue derived from the triple negative sample HCl-001 supported tumors that grew considerably faster than those from an ER/PR positive (HCI-011), an ER/PR and HER2-positive (HCI-005), or a HER2 positive (HCI-012) patient.

Immunohistochemistry of the triple negative tumor tissue (HCI-001) and the ER/PR-positive tissue (HCI-011) is shown in Fig 2C. This reveals that the status of CAIX and CAXII expression, along with ER status, was preserved across multiple transfers in tumor grafts. The marker for mitosis, Ki67, is significantly higher in tumors generated with HCI-001 tissue vs HCI-011 tissue (Fig 2C) in agreement with the rapid growth of tumors generated with the TNBC cells. Previous studies showed that CAIX expression in cancer-associated fibroblasts (CAFs) actually enhances expression of CAIX in PC3 prostate cells which drives the metastatic phenotype [37]. It is noteworthy that CAIX was not detected in the stromal tissue surrounding tumor cells in the immunohistochemistry images. To further explore the influence of CAFs on CAIX (or CAXII) expression in breast cancer cells, we co-cultured CAFs to represent stroma with UFH-001 and T47D cells (S3 Fig). The results indicate that the “mixing” of the environments of UFH-001 and T47D cells with CAFs (separately) does not influence the expression of CAIX or CAXII, in either breast cancer cells or CAFs. If CAIX (or CAXII) influence the pH of the tumor microenvironment, as has been hypothesized by us [35] and others [38–40], it does so through expression within the breast cancer cells themselves, and not through interactions with the tumor stroma.

### Cell-specific CA expression in breast cancer cells

**Fig 3:**
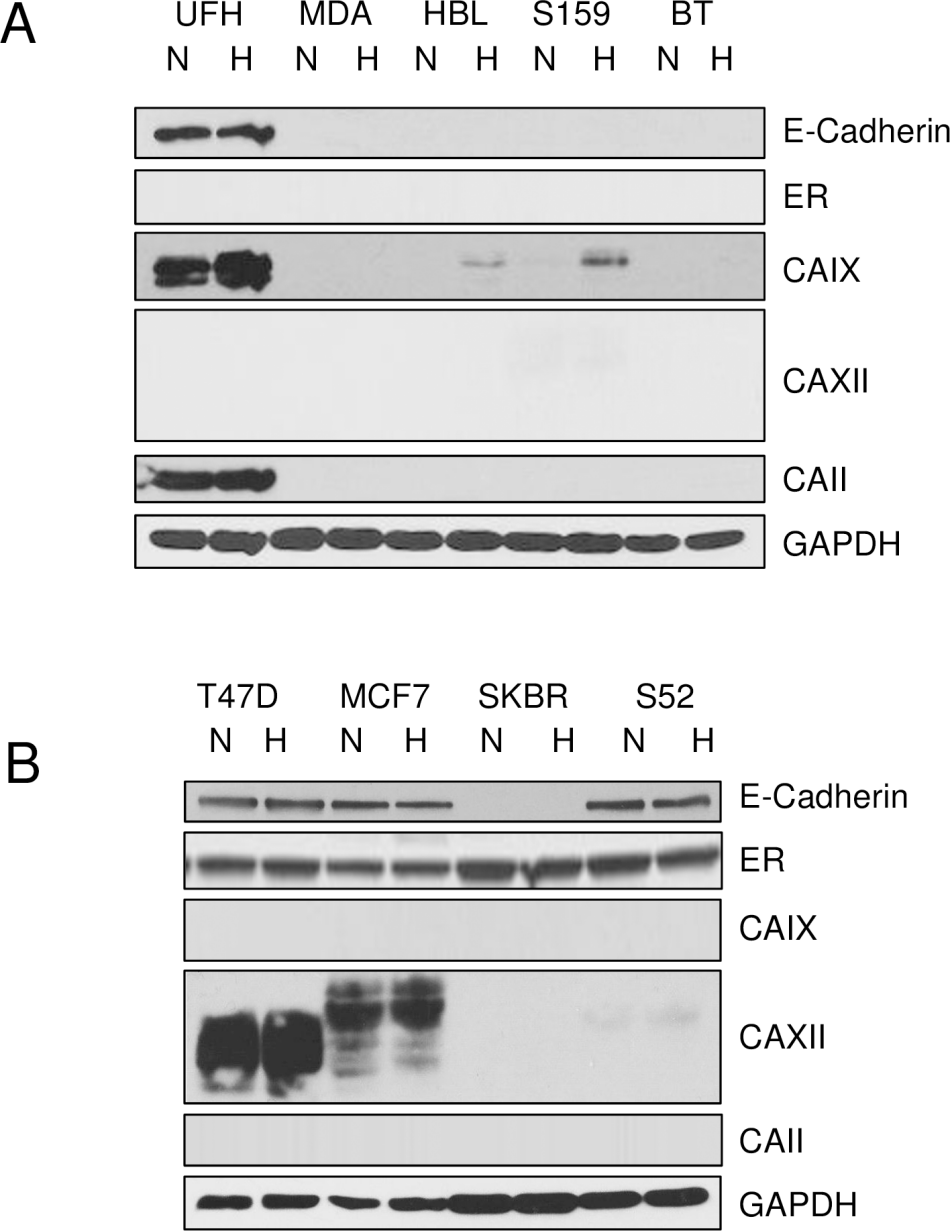
Differential expression of CAs in TNBC and luminal breast cancer cells. Cell were grown to 70% confluence and then exposed to normoxic (N) or 1% oxygen (H), hypoxic conditions. After 16 h, cells were washed with PBS and extracted in RIPA buffer containing protease inhibitors. Equal protein was loaded onto SDS PAGE gels, and then transferred to nitrocellulose for western blot analysis. Panel A. CA expression in TNBC cells: UFH = UFH-001, MDA = MDA-MB-231-LM2, HBL = HBL-100, S159 = Sum 159, BT = BT-549 cells. Panel B. CA expression in luminal breast cancer cells: T47D, MCF7 = MCF-7, SKBR = SKBR-3, S52 = SUM-52 cells.

In order to learn more about the function of CAIX and CAXII in breast cancer, we sought human breast cancer cell (hBrC) lines that replicated the tumor-specific expression of these proteins. In Fig 3, we demonstrate differential expression of CAIX and CAXII in TNBC and ER-positive breast cancer cells. Like the tumor grafts, not all TNBC breast cancer cells express CAIX, but none of the TNBC cells express CAXII (Fig 3A). Likewise, not all of the luminal lines express CAXII, yet none of them express CAIX (Fig 3B). This supports the idea that there is strong segregation of CAIX and CAXII expression in TNBC and luminal phenotypes, respectively. Surprisingly, the major cytosolic CA, CAII, was expressed only in the UFH-001 cells, a new TNBC line described in detail elsewhere [25]. Also surprising was the expression of E-cadherin in the UFH-001 cells (Fig 3A) which is normally a marker for the epithelial phenotype, as indicated in Fig 3B. However, E-cadherin expression has been observed in breast cancer cells at metastatic sites, suggesting that the “reappearance” of E-cadherin denotes a more aggressive phenotype [41]. Thus, the UFH-001 cells may represent these more aggressive cells that are able to colonize metastatic sites. These experiments also explored the hypoxic-dependent expression of the CAs. CAIX, but not CAXII (or CAII) was regulated by hypoxia.

### Knockdown of CAIX, but not CAXII, decreases proliferation

**Fig 4:**
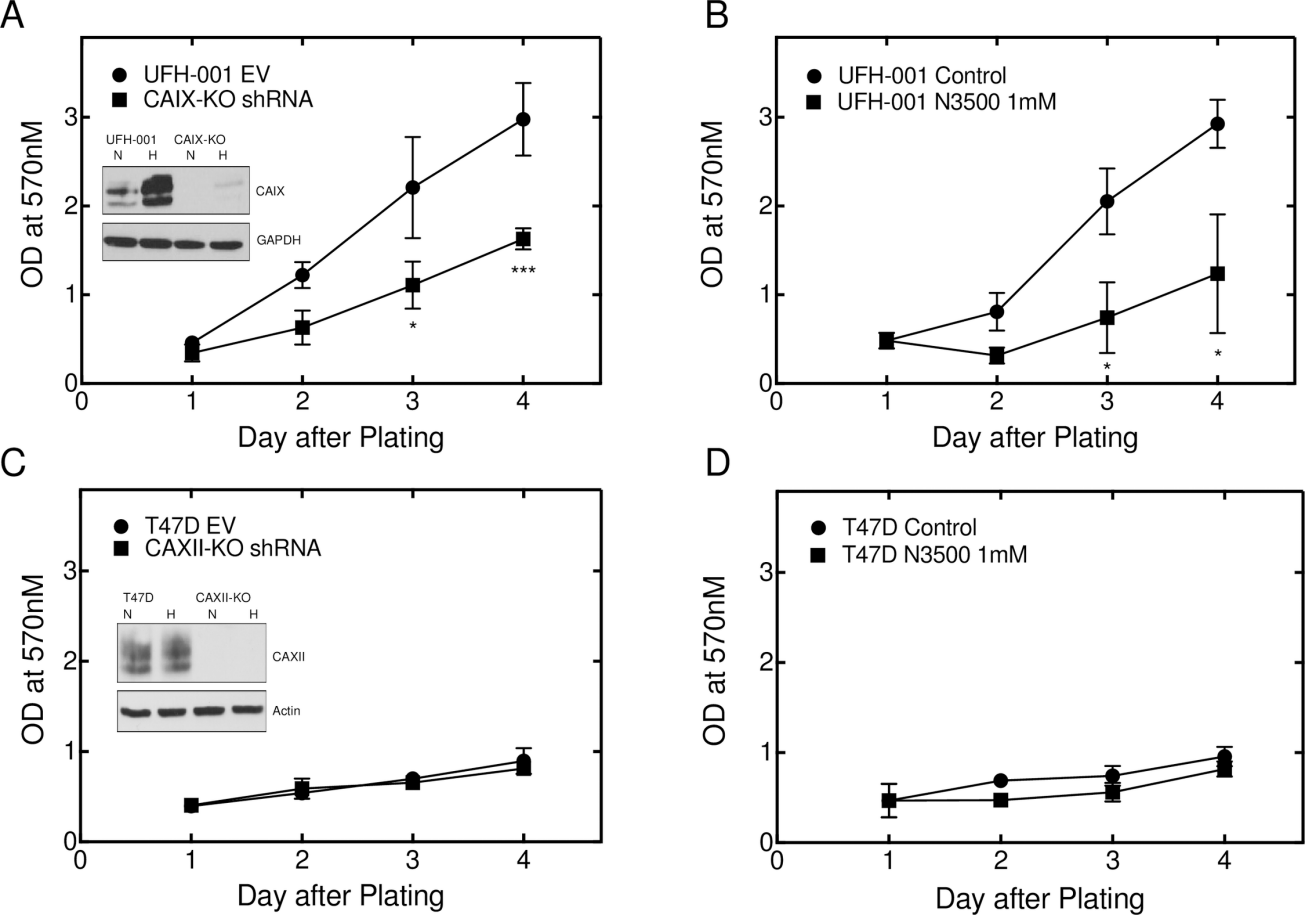
CAIX expression, but not that of CAXII, is important in breast cancer proliferation. Cell growth was monitored by the MTT assay in which CAIX and CAXII were knocked-down in UFH-001 and T47D cells using shRNA or activity was blocked using the impermeant sulfonamide inhibitor, N-3500 (1mM). Panels A and B. UFH-001 cells. Panels C and D. T47D cells. Asterisks denote statistical significance (* *p* < 0.05, *** *p* < 0.001.

We used an shRNA lentiviral strategy to knockdown CAIX expression in UFH-001 cells and CAXII in T47D cells (Fig 4). S1 Table shows the targeting sequences for both genes. CAIX expression in UFH-001 cells transfected with empty vector was compared with that of knockdown cells, exposed or not to hypoxia for 16 h (Fig 4A, insert). While CAIX expression fell below detectable levels in UFH-001 cells exposed to normoxic conditions, hypoxic cells showed some induced CAIX expression. Yet, cells in which CAIX was undetectable grew more slowly in culture than the empty vector control (Fig 4A). We also observed a significant reduction in growth of UFH-001 cells in culture in the presence of N-3500, an impermeant sulfonamide inhibitor of carbonic anhydrases whose characteristics are described elsewhere [34, 35] (Fig 4B). CAXII expression was completely ablated in T47D cells, whether or not exposed to hypoxia (Fig 4C, insert). However, knockdown of CAXII expression did not affect proliferation (Fig 4C), nor was the growth of T47D cells in culture sensitive to N-3500 (Fig 4D).

**Fig 5:**
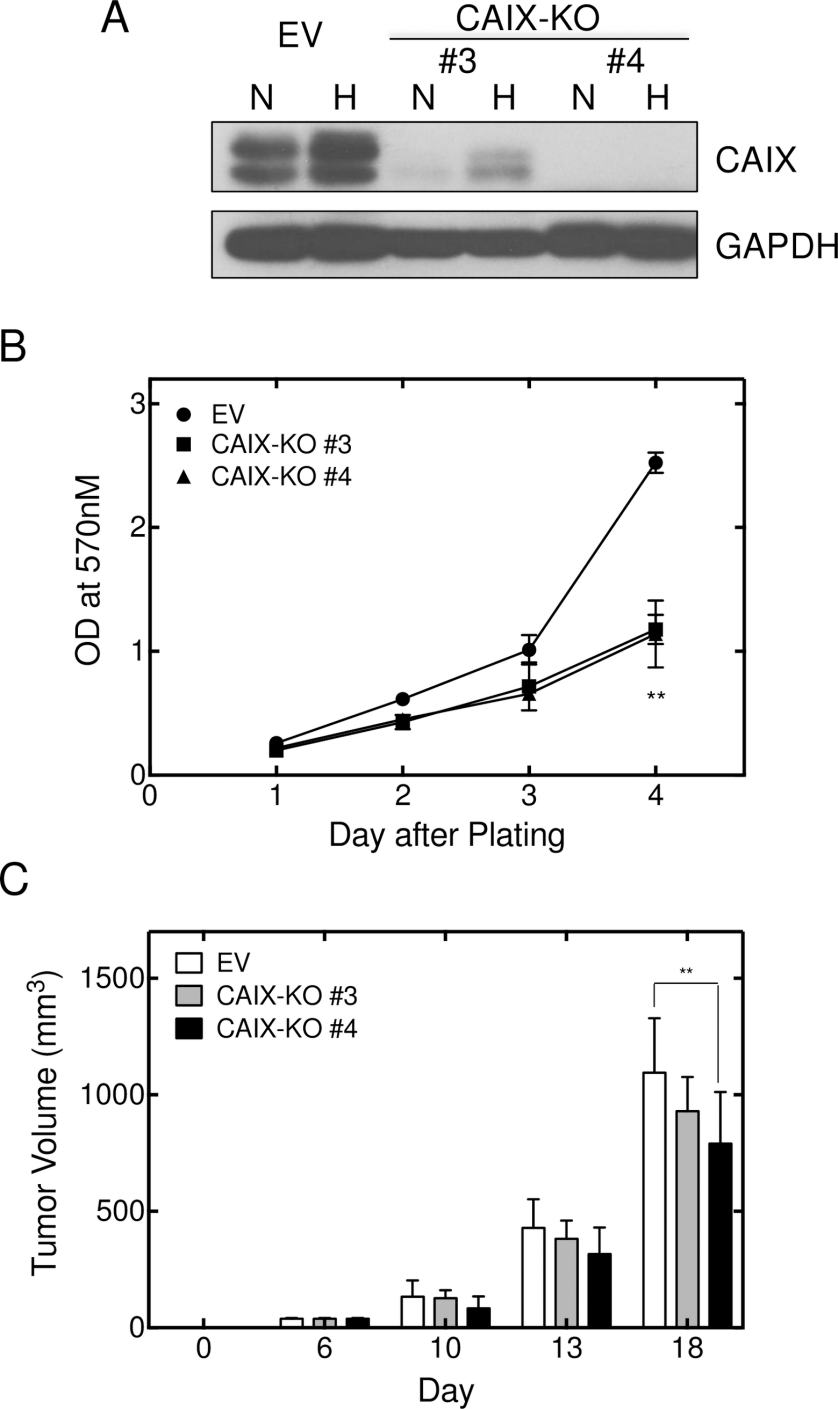
CAIX ablation decreases UFH-001 cell and tumor growth. CAIX was ablated using CRISPR/Cas9. Two probe sets are shown compared to the empty vector control. Panel A. Western blots were probed for CAIX and GAPDH. Panel B. Cell growth of knockdown cells and empty vector controls was evaluated using the MTT assay. Panel C. Orthotopically implanted cells (empty vector controls and knockdown cells) were monitored for tumor growth in NOD/SCID mice. Asterisks denote statistical significance (** *p* < 0.01).

**Fig 6:**
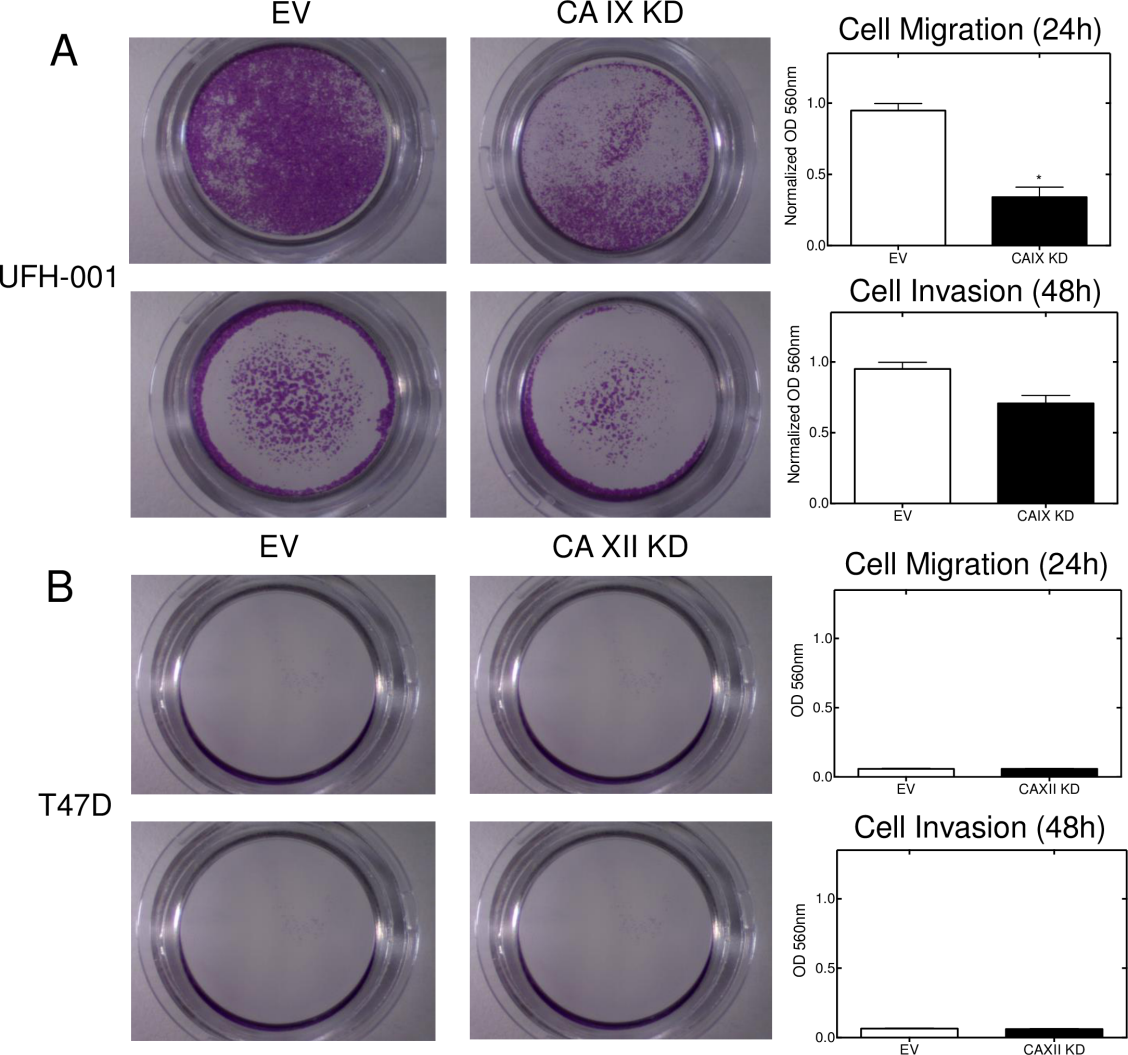
CAIX expression affects migration and invasion of breast cancer cells. Cell migration and invasion were determined using trans-well chambers. Panel A. UFH-001 cells (empty vector and CRISPR-CAIX knockdown cells from Fig 5) were plated in the upper transwell chambers and allowed to migrate or invade across the membrane for 24 h (upper images) or 48 h (lower images), respectively. Tabulation of results is shown to the right (*p* < 0.05). Panel B. T47D cells (empty vector or CAXII knockdown cells from Fig 4) were plated in the upper transwell chambers and allowed to migrate or invade across the membrane for 24 h (upper images) or 48 h (lower images), respectively. Tabulation of results is shown to the right.

We also used a CRISPR/CAS9 strategy for CAIX knockout in UFH-001 cells (Fig 5). We generated three clones using three independent sets of sgRNAs (S2 Table). One of those sets (#1) did not reduce CAIX expression (data not shown). Set 3# cells showed diminished expression of CAIX, while CAIX expression was undetectable in set #4 cells even under hypoxic conditions. In both cases, these were polyclonal populations which may explain the limited expression of CAIX in set #3 cells while set #4 cells represent complete knockout cells. Despite this difference in CAIX expression, both #3 and #4 cells exhibited reduced cell growth in culture compared to empty vector controls (Fig 5B). UFH-001 cells (controls and CRISPR knockouts) were then injected into mammary fat pads and evaluated for tumor growth (Fig 5C). Only RNA sgRNA #4 significantly reduced tumor growth compared to empty vector controls (*p* < 0.01), and only at the longest time point after implantation. We did not observe metastatic lesions under our conditions. We were unable to establish tumors with parental T47D cells (data not shown) so did not pursue xenografts with cells in which CAXII was ablated. Because the CRISPR strategy (sgRNA #4) was a complete knockout, we chose to use these cells in migration and invasion assays (Fig 6). These data show that CAIX knockdown led to a significant reduction in migration (*p* < 0.05) (Fig 6A) and a downward trend in invasion (Fig 6B), although not statistically significant (*p* = 0.08). T47D cells exhibited neither migratory nor invasive properties so the CAXII knockdown using cells were not informative in that regard.

### CA activity in TNBC and luminal breast cancer cells

**Fig 7:**
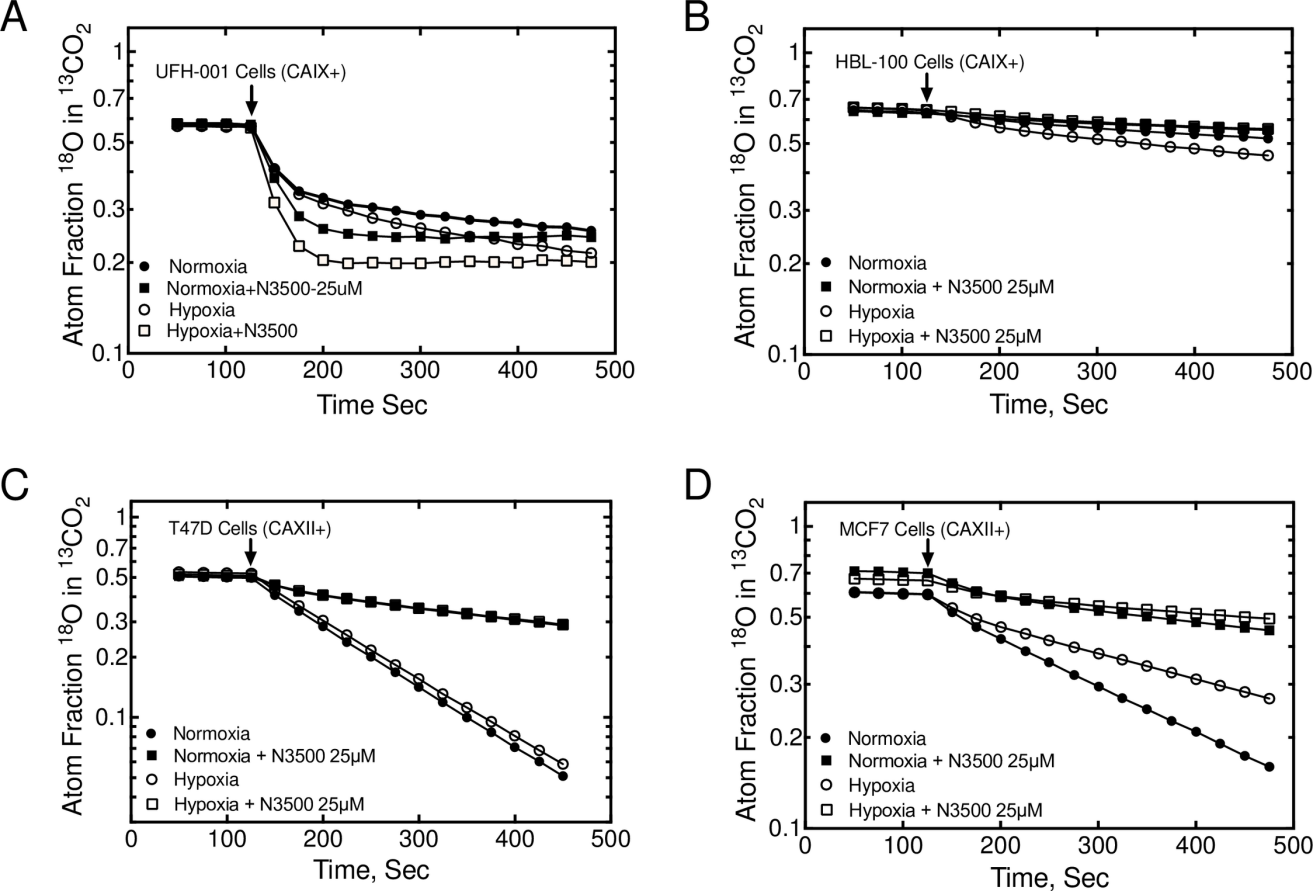
CAIX and CAXII activity in TNBC and luminal breast cancer cells. HBrCs were exposed to hypoxic conditions (or not) for 16 h. Cells were released form plates and suspended in bicarbonate-free medium and analyzed for carbonic anhydrase activity using the ^18^O exchange assay in the absence or presence of the impermeant sulfonamide inhibitor, N-3500. Data are representative of 3 independent biological replicates. Activity data for UFH-001 (Panel A), HBL-100 (Panel B), T47D (Panel C), and MCF7 (Panel D) cells are described in the text.

Because of the unique expression patterns of CAIX and CAXII in TNBC and luminal cells, respectively, this provided an opportunity to measure specifically the catalytic activity associated with these isoforms (Fig 7). Toward that end, we have employed the ^18^O exchange method to assess CA activity. The catalytic progress curve is observed as a loss of ^18^O from ^13^CO_2_, species, i.e., a negative slope. We chose two lines from each of our hBrC panels (see Fig 3). Because the UFH-001 cells express intracellular CAII activity, the progress curves are biphasic (Fig 7A). After addition of cells, the first phase (between ~ 130–180 sec into the progress curve) is representative of intracellular CAII activity [32]. The second phase (between 250–450 sec) measures exofacial CA activity [34, 42], in this case CAIX. The first order rate constants for the second phase of hypoxic versus normoxic cells differs by about 1.6-fold (1.17 x 10^-3^s^-1^ vs 0.70 x 10^-3^s^-1^), the small difference of which is related to the strong constitutive expression of CAIX at the time of assay (4 days after plating). To demonstrate specificity of this activity, we have used N-3500 to block exofacial CA activity [34, 35]. Activity in both normoxic and hypoxic cells was completely blocked (Fig 7A) in the presence of inhibitor. HBL-100 cells lack CAII, so the progress curves are linear which reflects only exofacial CAIX activity (Fig 7B). First order rate constants for hypoxic vs normoxic cells are 0.88x 10^-3^s^-1^ and 0.43 x 10^-3^s^-1^, respectively. These values are lower than those of UFH-001 cells, reflecting the reduced expression of CAIX between HBL-100 and UFH-001 cells (Fig 3A). Once again, N-3500 inhibited activity (Fig 7B). In the luminal T47D line, which also lacks CAII expression, the progress curves are also linear (Fig 7C). First order rate constants for hypoxic vs normoxic cells are 3.97 x 10^-3^s^-1^ and 4.32 x 10^-3^s^-1,^ respectively, reflecting CAXII activity, alone. This activity is significantly higher than that in UFH-001 (or HBL-100 cells), suggesting a higher level of expression of CAXII in T47D cells relative to CAIX in UFH-001 cells. Hypoxia did not enhance activity, but both normoxic and hypoxic cells were sensitive to inhibition by N-3500 (Fig 7C). Progress curves for MCF7 cells, which also express CAXII, were linear but revealed lower levels of activity than T47D cells (first order rate constants were 1.97 x 10^-3^s^-1^ vs 3.40 x 10^-3^s^-1^: hypoxia vs normoxia) (Fig 7D). In these cells, hypoxia actually decreased exofacial CA activity, even though CAXII expression did not appear to change (see Fig 3B). Again, the activity in both normoxic and hypoxic cells was inhibited by N-3500 (Fig 7D).

**Fig 8:**
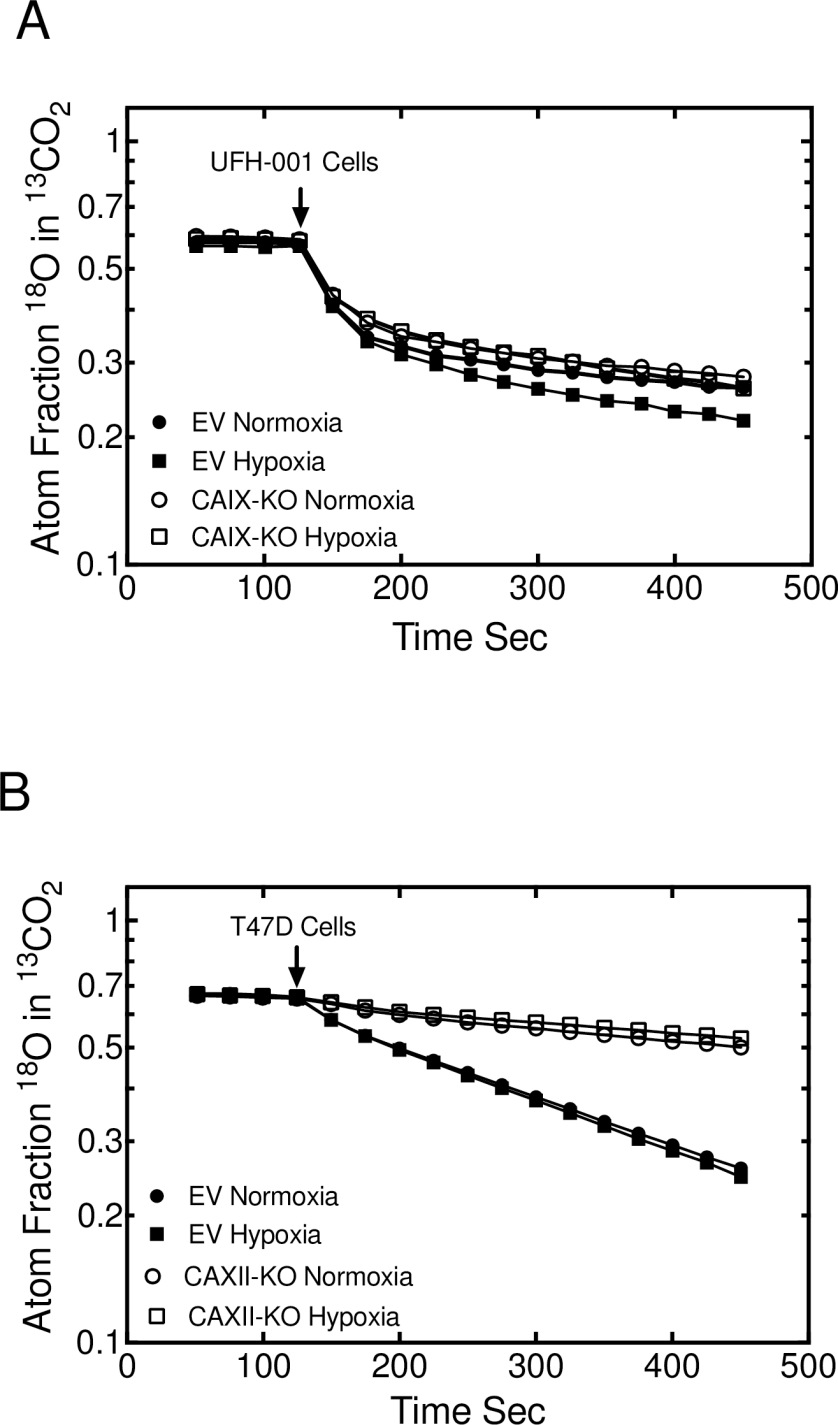
CA activity is reduced by ablation of CAIX and CAXII. ^18^O exchange activity was used to measure CA activity in empty vector controls and knockdown cells (from Fig 4) exposed to normoxic or hypoxic conditions. Panel A. UFH-001 cells. Panel B. T47D cells.

We also examined the effect of CAIX and CAXII knockdown (using the shRNA strategy) on CA activity in UFH-001 and T47D cells, respectively (Fig 8). In the empty vector UFH-001 cells, hypoxia increased catalytic activity compared to the normoxic empty vector control (Fig 8A), as in the parental line (Fig 7A). Surprisingly, only the hypoxic CAIX knockdown cells showed a decrease in catalytic activity. This contrasts to the strong effect of N-3500 on activity in parental UFH-001 (Fig 7A), where activity of both hypoxic and normoxic cells was reduced relative to controls. In addition, the shape of the progress curves were substantially different between the knockdown experiments and the N-3500 inhibited UFH-001 cells. Catalytic activity in CAXII-knockdown T47D cells is substantially reduced under both normoxic and hypoxic conditions (Fig 8B), resembling the effect of N-3500 on CA activity in the parental T47D cells (Fig 7C).

### CA activity increases in response to acidity

**Fig 9:**
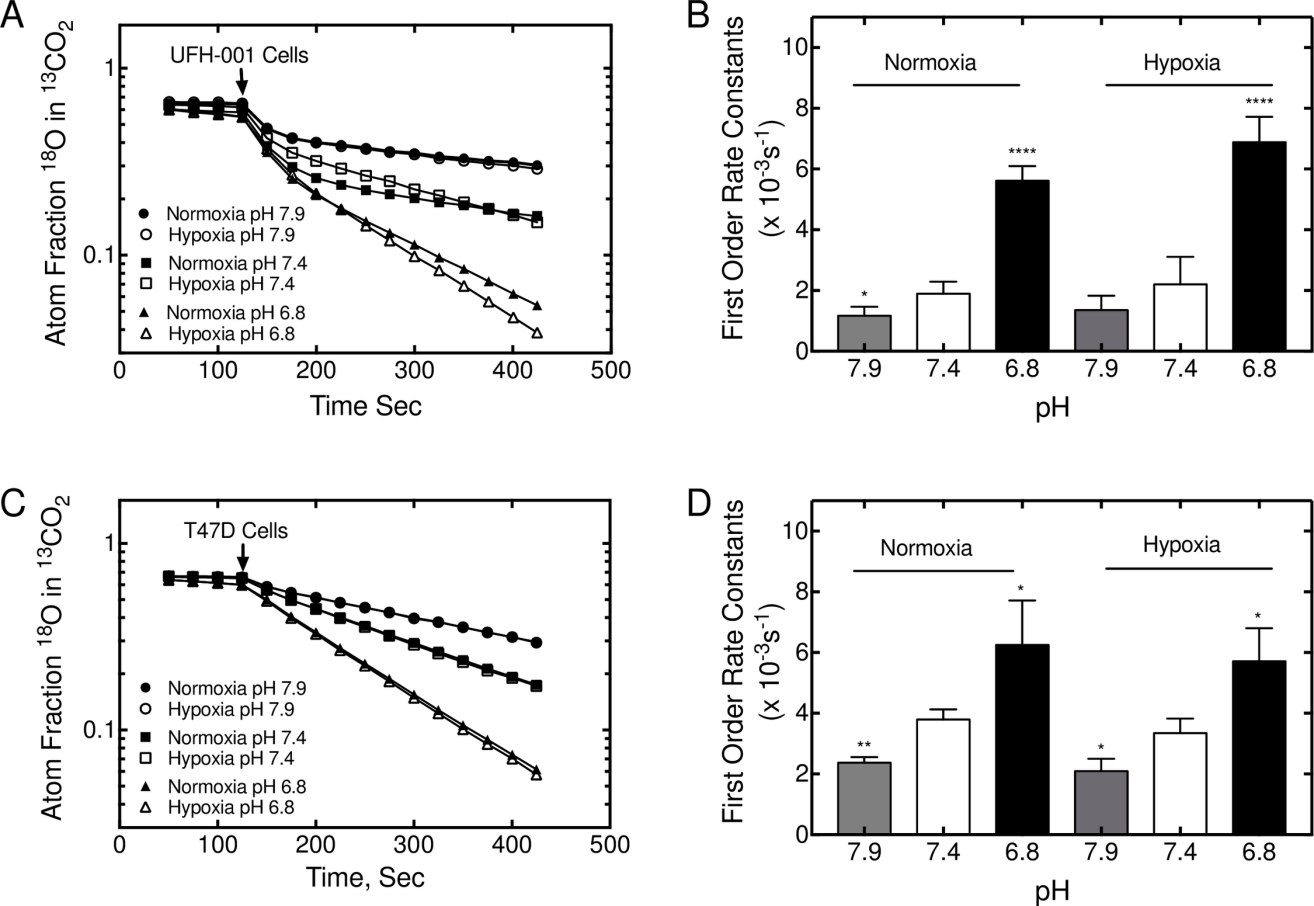
CA activity increases in response to low pH in UFH-001 and T47D cells. ^18^O exchange was used to measure CA activity in cells at pH 6.8, 7.4, and 7.9. Panels A and B. A representative plot of CAIX activity in response to pH is shown for UFH-001 cells exposed to normoxic or hypoxic conditions. Three independent biological replicates were used to quantify the first order rate constants. Panels C and D. A representative plot of CAXII activity in response to pH is shown for T47D cells exposed to normoxic and hypoxic conditions. Three independent biological replicates were used to quantify the first order rate constants. The asterisks denote statistical significance. * *p* < 0.05, ** *p* < 0.01, **** *p* < 0.0001.

Cancer cells exist in acidic microenvironments that can go as low as pH 6.5 [43, 44]. Here, we have evaluated the effect of pH on CA activity in UFH-001 and T47D cells (Fig 9). CAIX activity in normoxic UFH-001 cells increases by about 3-fold in response to an acute change in pH from 7.4 to 6.8 (Fig 9A and 9B). We also see a difference in activity in hypoxic cells between these two pH values, but detect an additional increase in activity reflecting the increase in CAIX protein. At pH 7.9, CAIX activity is equally reduced in normoxic and hypoxic UFH-001 cells relative to values determined at pH 7.4. CAXII activity in T47D cells also increases in response to reduced pH but is not responsive to hypoxia (Fig 9C and 9D). Once again, higher pH decreases CAXII activity.

### Catalytic activity of membrane associated with carbonic anhydrase

**Fig 10:**
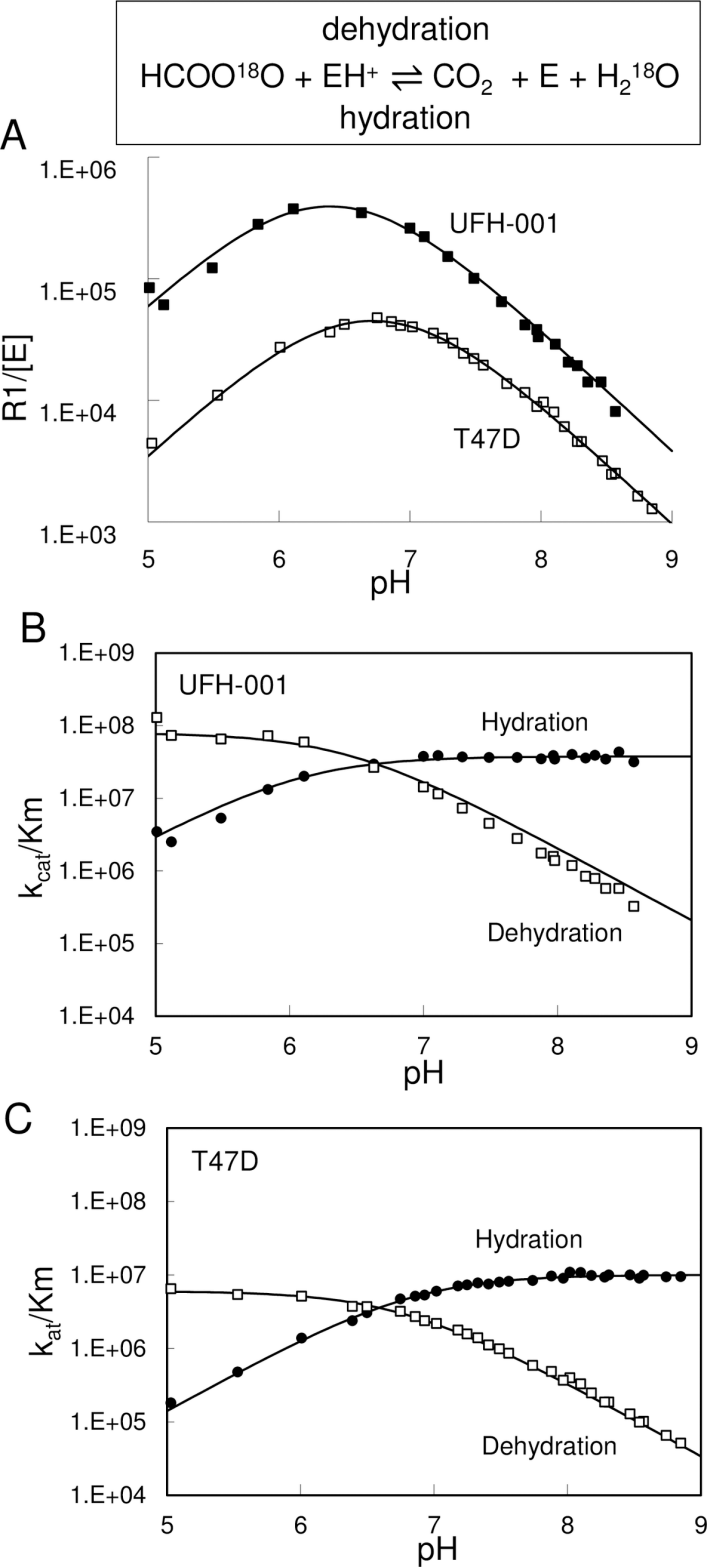
pH profile of activity by membrane-associated CAIX and CAXII. Membrane ghosts were prepared from hypoxic UFH-001 and normoxic T47D cells. Panel A. ^18^O exchange data is reported as the rate of activity divided by the enzyme concentration in a pH-dependent manner. The concentration of CAIX in the membrane suspension was estimated at 4.1 nM. The concentration of CAXII in the membrane suspension was estimated at 31.4 nM. Panel B. The kcat^exch^/K_eff_^s^ (kcat/Km) with units, M^-1^s^-1^, is shown for the hydration of CO_2_ and dehydration of bicarbonate catalyzed by CAIX. Panel C. the kcat^exch^/K_eff_^s^ (kcat/Km) with units, M^-1^s^-1^, is shown for the hydration of CO_2_ and dehydration of bicarbonate catalyzed by CAXII.

Carbonic anhydrases catalyze a reversible reaction. To determine the direction that is favored by reduced pH (i.e. hydration or dehydration), we isolated membrane ghosts from hypotonically-treated UFH-001 and T47D cells to perform pH titrations (Fig 10). CAIX showed higher catalytic activity per mole of enzyme over the entire pH range relative to CAXII, but especially at lower pH. The catalytic rate constants for the interconversion of CO_2_ and bicarbonate by CAIX and CAXII, R1/E, was determined directly from the rates of ^18^O depletion from bicarbonate (Fig 10A). Fitted data revealed a catalytic efficiency (kcat^exch^/K_eff_^CO^_2_) for CAIX of 3.8 x 10^7^ M^-1^s^-1^ with an apparent pKa of 6.2 ± 0.1 (n = 4) in the hydration direction, similar to the values we have previously calculated [35] and comparable to values determined for the recombinant CAIX catalytic domain [9, 45, 46]. For CAXII, the kcat^exch^/K_eff_^CO^_2_ was 1.0 x 10^7^ M^-1^s^-1^ with an apparent pKa of 7.0 ± 0.1 (n = 3). This is the first time that these values have been determined in isolated membranes, although these values are also similar to those published for the recombinant catalytic domain of CAXII [9, 45, 46]. The hydration reaction catalyzed by CAIX in UFH-001 membranes (favoring proton production) is less responsive to pH (Fig 10B) than is the hydration reaction in CAXII in T47D membranes (Fig 10C). By contrast, the dehydration reaction (favoring proton consumption) mediated by CAIX is significantly more sensitive to pH than that of CAXII. Overall, these data indicate that CAIX is more catalytically active than CAXII at low pH, an environment that typifies that of aggressive breast cancers.

## Discussion

Researchers often depend on mRNA expression patterns to address associations between gene transcription and disease states. Here, we show that across all types of breast cancer, elevated expression of CAIX mRNA is associated with lower patient survival compared to breast cancers with low expression (Fig 1A) but especially those with the TNBC phenotype (Fig 1B). In contrast, breast cancer patients whose tumors express high levels of CAXII mRNA have significantly better survival statistics than those with low expression of CAXII (Fig 1C). Surprisingly, the link between to ER-positive patients and CAXII expression at the mRNA level was not as impressive as expected (Fig 1D), based on the fact that CAXII protein is associated with the luminal phenotype [18–21] and is regulated by estrogen [18, 19]. Additional analysis mRNA expression in other subtypes (Supplemental data) revealed “mixed messages”. Here, high CAIX expression, associated with the luminal subtypes, suggested increased survival statistics while high CAXII expression in basal and HER2 positive breast cancers was associated with reduced survival statistics. This raised questions as to the value of mRNA expression, which may not reflect protein expression. In looking for model systems that could be utilized to examine the expression and function of CAIX and CAXII, individually but in the context of their native environments, we have now shown that CAIX and CAXII are differentially expressed across the subtypes of breast cancer using both PDXs (Fig 2) and cultured cells (Fig 3). In particular, these data confirm clinical data that CAIX protein expression is associated with the basal/TNBC phenotype while CAXII expression is associated with the luminal subtypes.

Because of the specificity of expression of CAIX and CAXII in TNBC and luminal breast cancer cells, we were able to further examine properties particular to CAIX and CAXII. These data show that CAIX, but not CAXII, plays some role in cell growth as only knockdown of CAIX leads to growth inhibition in cell culture (Figs 4A and 5B). Inhibition of CAIX activity also leads to cell growth reduction (Fig 4B). In addition, ablation of CAIX reduces the ability of UFH-001 cells to migrate in vitro (Fig 6) with a trend toward loss of invasiveness, implicating CAIX in these processes. Surprisingly, CAIX knockdown in the xenograft model (Fig 5C) shows only modest inhibition of tumor growth (CAIX-KO#4). The partial knock out, with greater than 90% loss of CAIX protein expression (CAIX-KO #3), showed no significant reduction in tumor volume compared to control. This appears to conflict with prior xenograft studies [47, 48]. Do our results bring into question CAIX as a therapeutic target? Based on our data, one could argue that any potential drug targeting CAIX might have to inhibit activity by nearly 100% to have an effect. While this is a difficult drug discovery hurdle, the strategy for designing new inhibitors include both greater efficacy and specificity. Yet, there are other explanations why we see only a small effect on tumor growth in CAIX-KO cells. The UFH-001 cell line is a very aggressive triple negative line, used specifically for its strong expression of CAIX. These cells expand so rapidly into xenograft tumors that we must sacrifice the mice earlier than indicated in other studies. So if the time of tumor burden could be extended, we might see greater inhibition of tumor growth. We also see strong central necrosis, suggesting the signals emanating from these tumors to activate angiogenesis is insufficient. While we did not observe metastatic lesions from the primary tumor, we do see brain metastases when cells are injected under the front arm and lymphatic invasion when injected into the tail vein (unpublished data). We are currently testing whether CAIX ablation affects these metastatic events. Perhaps CAIX ablation will have a greater affect on metastasis compared to tumor growth. Indeed, it has been suggested that CAIX is part of the migration apparatus in lamellipodia serving as a “catalyst” for cell migration [49]. These structures are interesting because they exhibit intense glycolysis in the absence of mitochondria at the leading edge of the cell in motion [50, 51]. In this context, CAIX is likely to participate in a functional metabolon (along with AE2, NHE1 and CAII) that leads to pH regulation of both the intra and extracellular space [52–55].

In UFH-001 cells, knockdown of CAIX reduces CA activity in hypoxic cells (Fig 8) although the loss of activity is significantly less than in the presence of the inhibitor, N-3500 (Fig 7). Yet, inhibition of catalytic activity also reduces UFH-001 cell growth (Fig 4B), even though high concentrations of the N-3500 inhibitor (1 mM) are required relative to the Ki value for inhibition of activity (12.6 ± 3.3 μM [35]). One explanation for this disconnect between growth and activity could be the presence of another CA isoform. However, there is no CAXII in UFH-001 cells, and little evidence that either of the other membrane bound CAs, CAIV or CAXIV, is expressed in breast cancer cells [56, 57]. An alternative possibility could be that CAIX has other properties that could regulate cell growth independent of its activity. In this context, there is evidence that the intracellular tail is critical to CAIX function [58]. Specifically, Thr 443 in the cytoplasmic tail is susceptible to phosphorylation by PKA [59]. This could lead to down-stream signaling that may be important for CAIX-dependent cell growth. This is further supported by our observation that UFH-001 cells lacking expression of CAIX (CRISPR strategy) reduce tumor growth compared to empty vector controls, but again not to the expected extent based on published data in other TNBC xenograft models as discussed above [47]. By comparison, proliferation of T47D cells is unaffected by N-3500, although activity is completely blocked by N-3500 and knockdown of CAXII. CAXII does not have a consensus sequence for PKA-mediated phosphorylation, which perhaps implicates further a PKA-dependent signaling system in CAIX-regulated cell growth.

Both CAIX activity (in UFH-001 cells) and CAXII activity (in T47D cells) are sensitive to pH. This is expected for a bicarbonate-based reaction. At low pH, the activity of CAIX and CAXII (in cells) increases but experiments with membrane ghosts reveal that the dehydration activity (consumption of protons) drives the increase in overall activity. And clearly, the dehydration activity of CAIX is significantly higher than that of CAXII. Further, the dehydration activity of CAIX continues to increase below pH 6.8, relative to CAXII, which is typical of pH in the tumor microenvironment. This may be due in part to the stability of CAIX relative to CAXII at low pH [60]. Together, these data suggest that CAIX is more efficient than CAXII at low pH and that it shifts the equilibrium between CO_2_ and bicarbonate to favor CO_2_ production (and proton consumption). This may provide a mechanism for stabilizing pH at values in the tumor microenvironment of TNBC that favor cancer cell survival.

CAIX and CAXII expression are diagnostic for breast cancer subtype and prognosticators of patient survival. Overall, we show that CAIX but not CAXII drives growth, migration, and metastasis consistent with its expression in more aggressive breast cancers. That they are affected by pH in such different ways may underlie the properties of these proteins. While the work with breast cancer cells has allowed an appreciation of these differences, the PDX models may ultimately provide more physiological relevance in understanding the role of CAIX and CAXII in the context of breast cancer. Future studies using PDX models combined with CA inhibitors should be revealing in differentiating the role of CAIX and CAXII *in vivo*. Because CAXII is found in normal breast tissue, new inhibitors with higher efficacy for CAIX vs CAXII may allow specific targeting to avoid losing the potential beneficial effects of CAXII.

## Supporting information

S1 Fig Role of CA9 gene expression in breast cancer patient survival. mRNA from breast cancer patients (unrestricted analysis) was probed for the *CA9* gene expression (CAIX-mRNA) using Affimetrix ID 205199_at. **Panel A** represents the data from Basal breast cancers; **Panel B** represents data from HER2 positive breast cancers; **Panel C** represents Luminal A breast cancers; and **Panel D** represents Luminal B breast cancers.
(PPTX)

S2 Fig Role of CA12 gene expression in breast cancer patient survival. mRNA from breast cancer patients (unrestricted analysis) was probed for the *CA12* gene expression (CAIX-mRNA) using Affimetrix ID 215867_at. **Panel A** represents the data from Basal breast cancers; **Panel B** represents data from HER2 positive breast cancers; **Panel C** represents Luminal A breast cancers; and **Panel D** represents Luminal B breast cancers.
(PPTX)

S3 Fig Co-culture of breast cancer cells with cancer-associated fibroblasts. Cancer-associate fibroblasts (CAFs) were first plated in 6-well plates at a density of 3x10^5^ cells/well. Within 1 hour of CAFs plating, UFH-001 (empty vector or CAIX-KO cells) or T47D cells (empty vector or CAXII-KO cells) were plated on 6-well Trans-well inserts (0.4um) at a density of 3x10^4^ cell/insert and 6x10^4^ cell/insert respectively. CAFs and breast cancer cells were then co-cultured at 37°C in 5% CO_2_ for 5 days. Cells were lysed and analyzed for protein expression by western blot analysis. **Panel A**. Extracts from normoxic (N) or hypoxic (H) UFH-001 cells (empty vector or CAIX-KKO) were probed for CAIX or GAPDH expression in the absence or presence (+) of CAFs. **Panel B**. Extracts from CAF cells, co-cultured or not with UFH-001 cells (empty vector or CAIX KO) under normoxic (N) or hypoxic (h) conditions, were probed for CAIX or GAPDH expression. **Panel C**. Extracts from normoxic (N) or hypoxic (H) T47D cells (empty vector or CAXII KO) were probed for CAXII or GAPDH expression in the absence or presence (+) of CAFs. **Panel D**. Extracts from CAF cells, co-cultured with T47D cells (empty vector or CAXII-KO) under normoxic (N) or hypoxic (H) conditions, were probed for CAXII or GAPDH expression.
(PPTX)

S1 Table Gene targeting sequences used in GIPZ lentiviral shRNA particles. Clone ID and gene targeting sequences are provided for construction of lentivirus shRNA particles to deplete expression of the *CA9* (CAIX-mRNA) and *CA12* (CAXII-mRNA)
(PPTX)

S2 Table Primer sequences for guide RNA expression plasmids for CAIX knockout. Clone ID and gene targeting sequences are provided for crispr knockout of the *CA9 gene* (CAIX-mRNA).
(PPTX)
