# Expression of Concern: Differential expression and function of CAIX and CAXII in breast cancer: A comparison between tumorgraft models and cells

**DOI:** 10.1371/journal.pone.0327518

**Published:** 2025-07-03

**Authors:** The PLOS One Editors

After this article [1] was published, concerns were raised regarding results presented in Figs 4 and 6. Specifically:

- The western blot inserts in Figs 4A and C in [1] appear similar to Figs S4A and B in [2].
- The following panels appear similar:
  - All four panels of Fig 6B in [1] and the MCF 10A panels of Figs 6B and 6C in [3].
  - Fig 6A Migration EV in [1], Fig 9A Migration NC in [2], Fig 9A Migration 10 μM U-CH_3_ in [2], and Fig 6B UFH-001 in [3].
  - Fig 6A Invasion EV in [1] and Fig 9A Invasion 100 μM U-CH_3_ in [2].
  - Fig 6A Invasion CA IX KD in [1] and Fig 9A Invasion 10 μM U-F in [2].
  - Fig 6A Migration CA IX KD in [1] and Fig 9A Migration 100 μM U-NO_2_ in [2].

Regarding the western blot concerns in Fig 4, the corresponding author stated that the western blots in Figs 4A and C in [1] were intentionally reused in Figs S4A and B in [2], and that there is no quantitative connection between the western blots in Figs 4A and C and the growth data across Figs 4A-D. For the plate image concerns in Fig 6, the corresponding author stated that the images in Fig 9A in [2] are correct and the images in Figs 6A and B in [1] are incorrect. An updated version of Fig 6 with the correct panels from the original experiments is provided here. The underlying images for the corrected Fig 6 are in S5-S12 Files, and the associated underlying data for the charts in Fig 6 are in S4 File.

**Fig 6:**
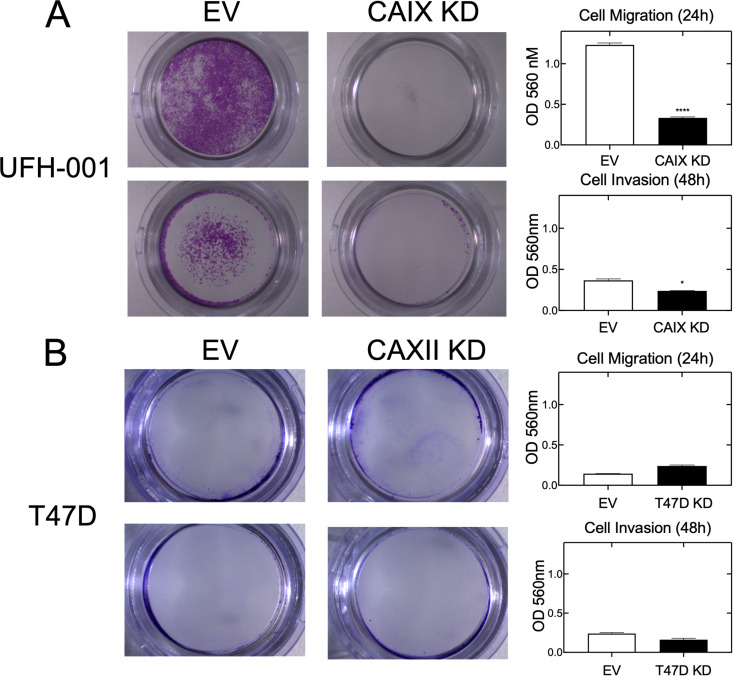
CAIX expression affects migration and invasion of breast cancer cells. Cell migration and invasion were determined using trans-well chambers. Panel A. UFH-001 cells (empty vector and CRISPR-CAIX knockdown cells from Fig 5) were plated in the upper transwell chambers and allowed to migrate or invade across the membrane for 24 h (upper images) or 48 h (lower images), respectively. Tabulation of results is shown to the right (*p* < 0.05). Panel B. T47D cells (empty vector or CAXII knockdown cells from Fig 4) were plated in the upper transwell chambers and allowed to migrate or invade across the membrane for 24 h (upper images) or 48 h (lower images), respectively. Tabulation of results is shown to the right.

With the exception of the underlying data for Figs 2B, 5B and 7A and the western blots, which the corresponding author stated are no longer available, the underlying data for the remainder of this article [1] are provided here in S1-S3 and S14-S24 Files. Analysis of a different data set from a later repeat experiment of Fig 7A under the same experimental conditions is provided here in S13 File. The corresponding author stated that the quantitative data for Fig 1 were generated using the Meyer Kaplan database [4].

Given that the primary data underlying Figs 2B, 5B and 7A are [1] are no longer available, and the primary data were not provided with the published article [1], contrary to the Data Availability statement, this article [1] does not comply with the PLOS Data Availability Policy.

In light of the concerns listed above, and as this article [1] does not comply with the PLOS Data Availability Policy, the *PLOS One* Editors issue this Expression of Concern.

Owing to the concerns about similarities with previously published content [3], published 2018 Taylor & Francis which is not offered under a CC BY license, the Migration EV panel of Fig 6A and all four panels of Fig 6B are excluded from this article’s [1] license.

## Supporting information

S1 File Underlying quantitative data in support of the charts in Figs 4A and C in [1]. (XLSX)

S2 File Underlying quantitative data in support of the chart in Fig 4B in [1]. (XLSX)

S3 File Underlying quantitative data in support of the chart in Fig 4D in [1]. (XLSX)

S4 File Underlying quantitative data in support of the charts in Fig 6 in [1]. (XLSX)

S5 File Underlying image for the corrected Fig 6A UFH-001 EV migration panel. (TIFF)

S6 File Underlying image for the corrected Fig 6A UFH-001 CAIX migration panel. (TIF)

S7 File Underlying image for the corrected Fig 6A UFH-001 EV invasion panel. (TIFF)

S8 File Underlying image for the corrected Fig 6A UFH-001 CAIX invasion panel. (TIF)

S9 File Underlying image for the corrected Fig 6B T47D EV migration panel. (JPG)

S10 File Underlying image for the corrected Fig 6B T47D CAXII KD migration panel. (JPG)

S11 File Underlying image for the corrected Fig 6B T47D EV invasion panel. (JPG)

S12 File Underlying image for the corrected Fig 6B T47D CAXII KD invasion panel. (JPG)

S13 File Prism/graph pad file with replicate data and figure in support of Fig 7A. This represents an analysis of a different data set to Fig 7A in [1] with the same experimental conditions.
(PRISM)

S14 File Prism/graph pad file with original data and figure in support of Fig 7B. (PZFX)

S15 File Prism/graph pad file with original data and figure in support of Fig 7C.
(PRISM)

S16 File Prism/graph pad file with original data and figure in support of Fig 7D. (PZFX)

S17 File Prism file with original data and figure in support of Fig 8A. (PRISM)

S18 File Prism file with original data and figure in support of Fig 8B. (PZFX)

S19 File Prism file with quantitative data and figure in support of Fig 9C. (PZFX)

S20 File Prism file with quantitative data and figure in support of Fig 9D. (PZFX)

S21 File Prism file with quantitative data and figure in support of Fig 9A. (PZFX)

S22 File Prism file with quantitative data and figure in support of Fig 9B. (PZFX)

S23 File Underlying quantitative data in support of Fig 10 in [1]. (XLS)

S24 File Underlying quantitative data in support of Fig 5C in [1].
(XLSX)
