# Supplementary material for: Differential expression and function of CAIX and CAXII in breast cancer: A comparison between tumorgraft models and cells
Source: PLoS One. 2018 Jul 2;13(7):e0199476. doi: 10.1371/journal.pone.0199476 (PMC6028082; doi:10.1371/journal.pone.0199476)
Supplement: S1 Fig — mRNA from breast cancer patients (unrestricted analysis) was probed for the CA9 gene expression (CAIX-mRNA) using Affimetrix ID 205199_at. Panel A represents the data from Basal breast cancers; Panel B represents data from HER2 positive breast cancers; Panel C represents Luminal A breast cancers; and Panel D represents Luminal B breast cancers. (PPTX) [file pone.0199476.s001.pptx]

## Slide 1
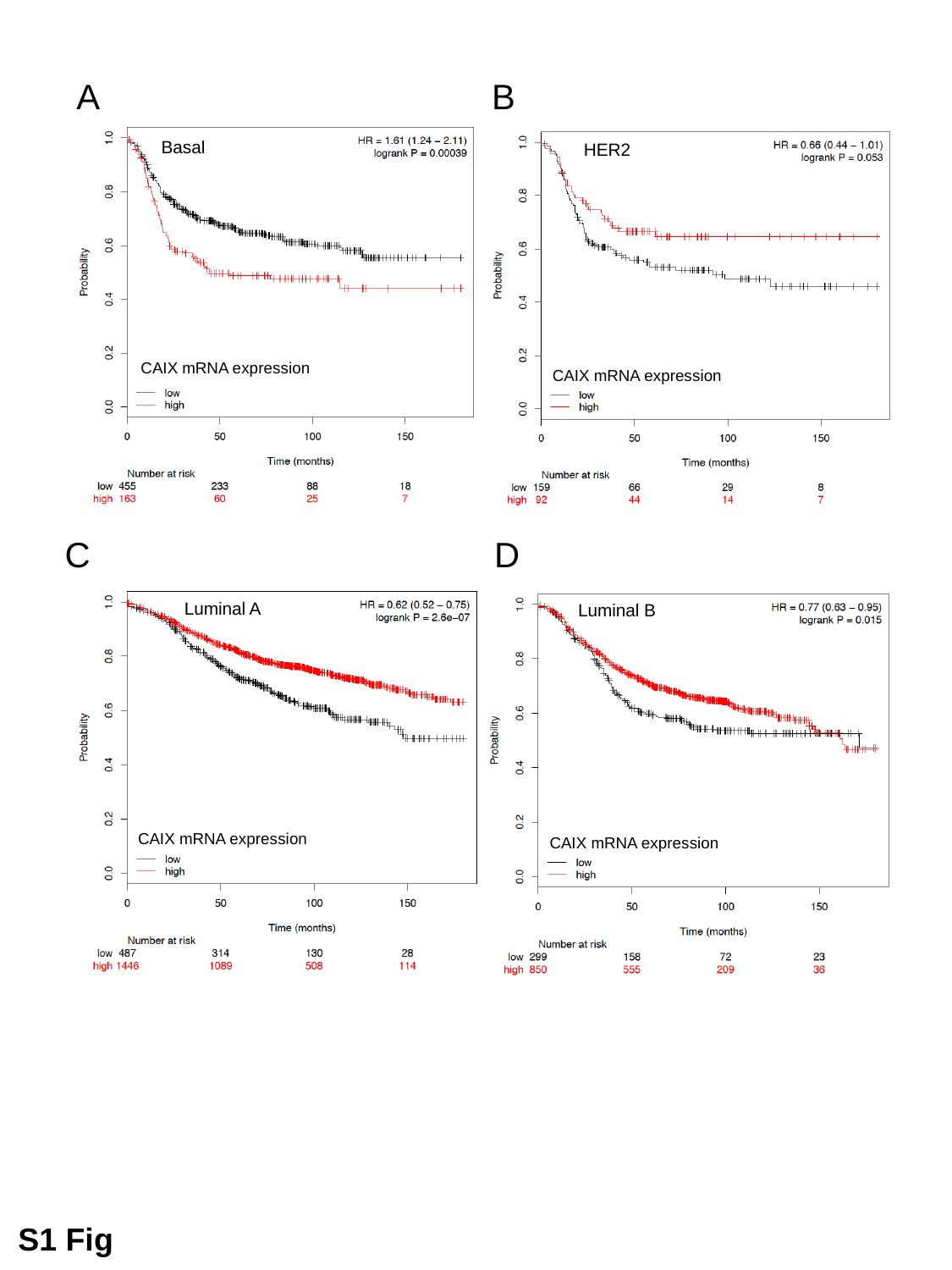

A
B
Basal
CAIX mRNA expression
HER2
CAIX mRNA expression
C
D
Luminal B
CAIX mRNA expression
Luminal A
CAIX mRNA expression
S1 Fig
