# Supplementary material for: Differential expression and function of CAIX and CAXII in breast cancer: A comparison between tumorgraft models and cells
Source: PLoS One. 2018 Jul 2;13(7):e0199476. doi: 10.1371/journal.pone.0199476 (PMC6028082; doi:10.1371/journal.pone.0199476)
Supplement: S2 Fig — mRNA from breast cancer patients (unrestricted analysis) was probed for the CA12 gene expression (CAIX-mRNA) using Affimetrix ID 215867_at. Panel A represents the data from Basal breast cancers; Panel B represents data from HER2 positive breast cancers; Panel C represents Luminal A breast cancers; and Panel D represents Luminal B breast cancers. (PPTX) [file pone.0199476.s002.pptx]

## Slide 1
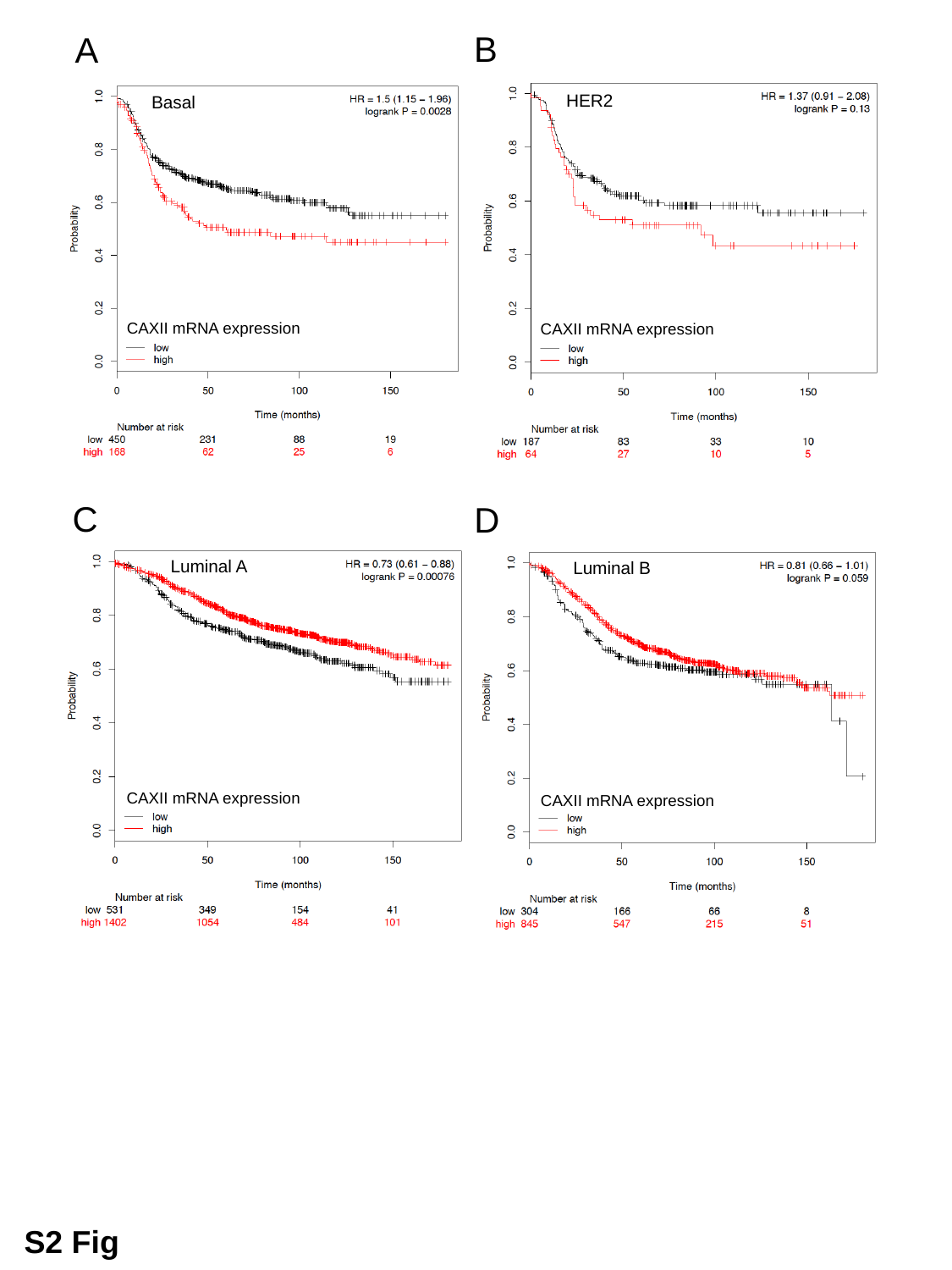

B
A
Basal
CAXII mRNA expression
HER2
CAXII mRNA expression
C
D
Luminal A
CAXII mRNA expression
Luminal B
CAXII mRNA expression
S2 Fig
