# Supplementary material for: Differential expression and function of CAIX and CAXII in breast cancer: A comparison between tumorgraft models and cells
Source: PLoS One. 2018 Jul 2;13(7):e0199476. doi: 10.1371/journal.pone.0199476 (PMC6028082; doi:10.1371/journal.pone.0199476)
Supplement: S3 Fig — Cancer-associate fibroblasts (CAFs) were first plated in 6-well plates at a density of 3x10^5^ cells/well. Within 1 hour of CAFs plating, UFH-001 (empty vector or CAIX-KO cells) or T47D cells (empty vector or CAXII-KO cells) were plated on 6-well Trans-well inserts (0.4um) at a density of 3x10^4^ cell/insert and 6x10^4^ cell/insert respectively. CAFs and breast cancer cells were then co-cultured at 37°C in 5% CO2 for 5 days. Cells were lysed and analyzed for protein expression by western blot analysis. Panel A. Extracts from normoxic (N) or hypoxic (H) UFH-001 cells (empty vector or CAIX-KKO) were probed for CAIX or GAPDH expression in the absence or presence (+) of CAFs. Panel B. Extracts from CAF cells, co-cultured or not with UFH-001 cells (empty vector or CAIX KO) under normoxic (N) or hypoxic (h) conditions, were probed for CAIX or GAPDH expression. Panel C. Extracts from normoxic (N) or hypoxic (H) T47D cells (empty vector or CAXII KO) were probed for CAXII or GAPDH expression in the absence or presence (+) of CAFs. Panel D. Extracts from CAF cells, co-cultured with T47D cells (empty vector or CAXII-KO) under normoxic (N) or hypoxic (H) conditions, were probed for CAXII or GAPDH expression. (PPTX) [file pone.0199476.s003.pptx]

## Slide 1
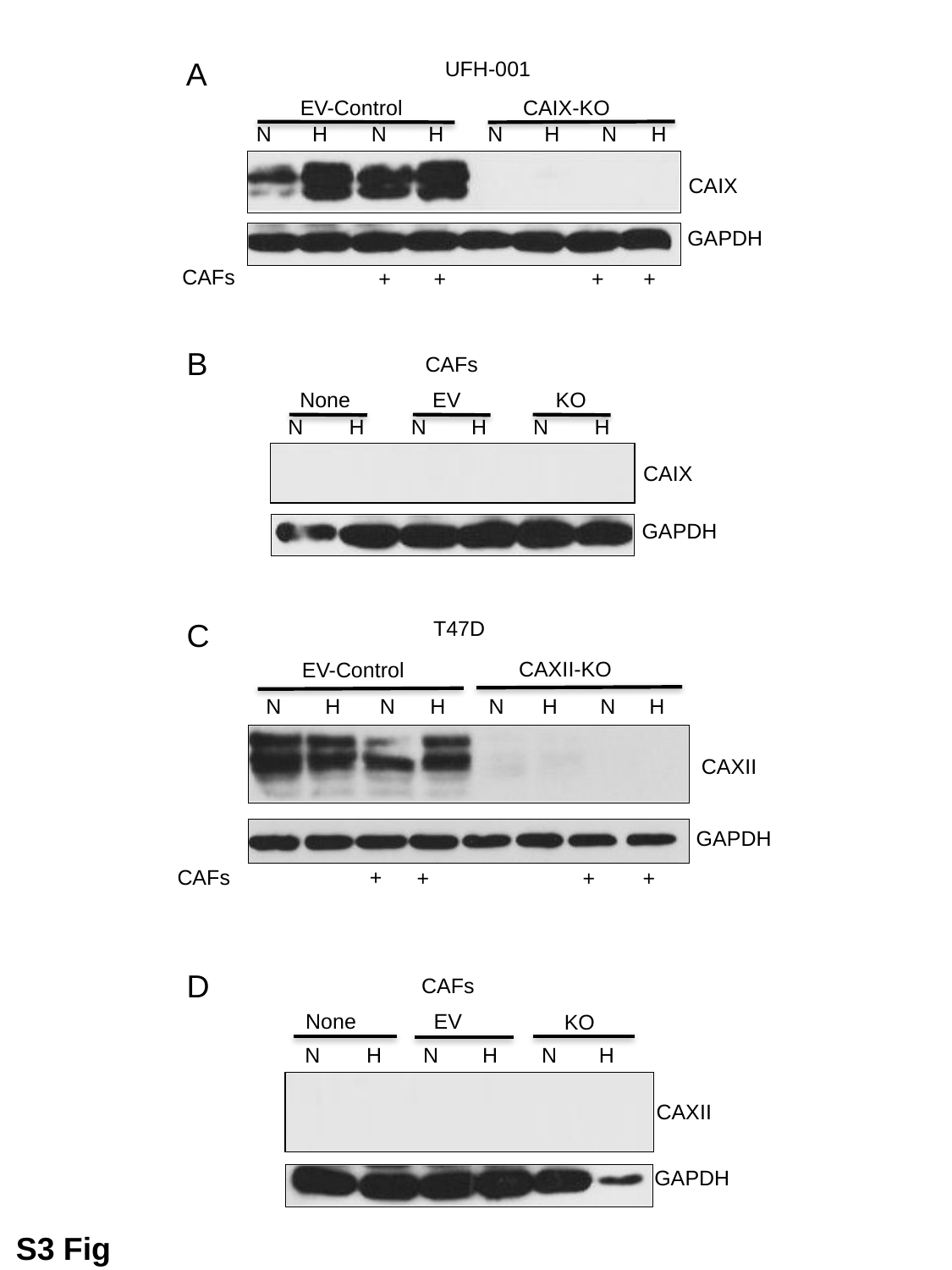

A
UFH-001
EV-Control
CAIX-KO
N
H
N
H
N
H
N
H
CAFs
+
+
+
+
CAIX
GAPDH
B
None
EV
KO
N
H
N
H
N
H
CAFs
CAIX
GAPDH
T47D
CAXII-KO
EV-Control
N
N
H
H
N
H
H
N
CAFs
+
+
+
+
C
CAXII
GAPDH
D
CAFs
EV
None
KO
N
H
N
H
N
H
CAXII
GAPDH
S3 Fig
