# Supplementary material for: Differential expression and function of CAIX and CAXII in breast cancer: A comparison between tumorgraft models and cells
Source: PLoS One. 2018 Jul 2;13(7):e0199476. doi: 10.1371/journal.pone.0199476 (PMC6028082; doi:10.1371/journal.pone.0199476)
Supplement: S1 Table — Clone ID and gene targeting sequences are provided for construction of lentivirus shRNA particles to deplete expression of the CA9 (CAIX-mRNA) and CA12 (CAXII-mRNA) (PPTX) [file pone.0199476.s004.pptx]

## Slide 1
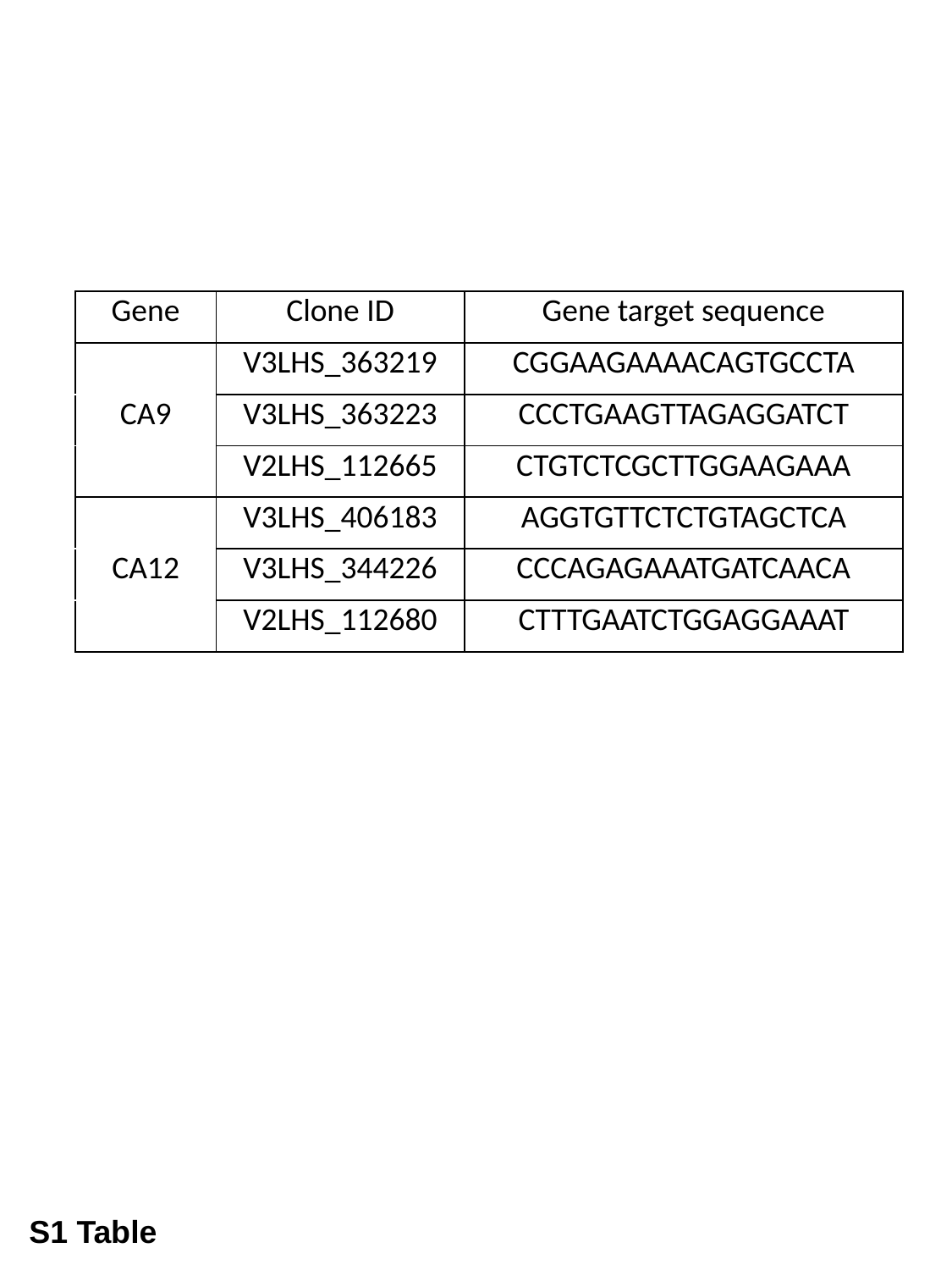

| Gene | Clone ID | Gene target sequence |
| --- | --- | --- |
| | V3LHS\_363219 | CGGAAGAAAACAGTGCCTA |
| CA9 | V3LHS\_363223 | CCCTGAAGTTAGAGGATCT |
| | V2LHS\_112665 | CTGTCTCGCTTGGAAGAAA |
| | V3LHS\_406183 | AGGTGTTCTCTGTAGCTCA |
| CA12 | V3LHS\_344226 | CCCAGAGAAATGATCAACA |
| | V2LHS\_112680 | CTTTGAATCTGGAGGAAAT |
S1 Table
