# Supplementary material for: Differential expression and function of CAIX and CAXII in breast cancer: A comparison between tumorgraft models and cells
Source: PLoS One. 2018 Jul 2;13(7):e0199476. doi: 10.1371/journal.pone.0199476 (PMC6028082; doi:10.1371/journal.pone.0199476)
Supplement: S2 Table — Clone ID and gene targeting sequences are provided for crispr knockout of the CA9 gene (CAIX-mRNA). (PPTX) [file pone.0199476.s005.pptx]

## Slide 1
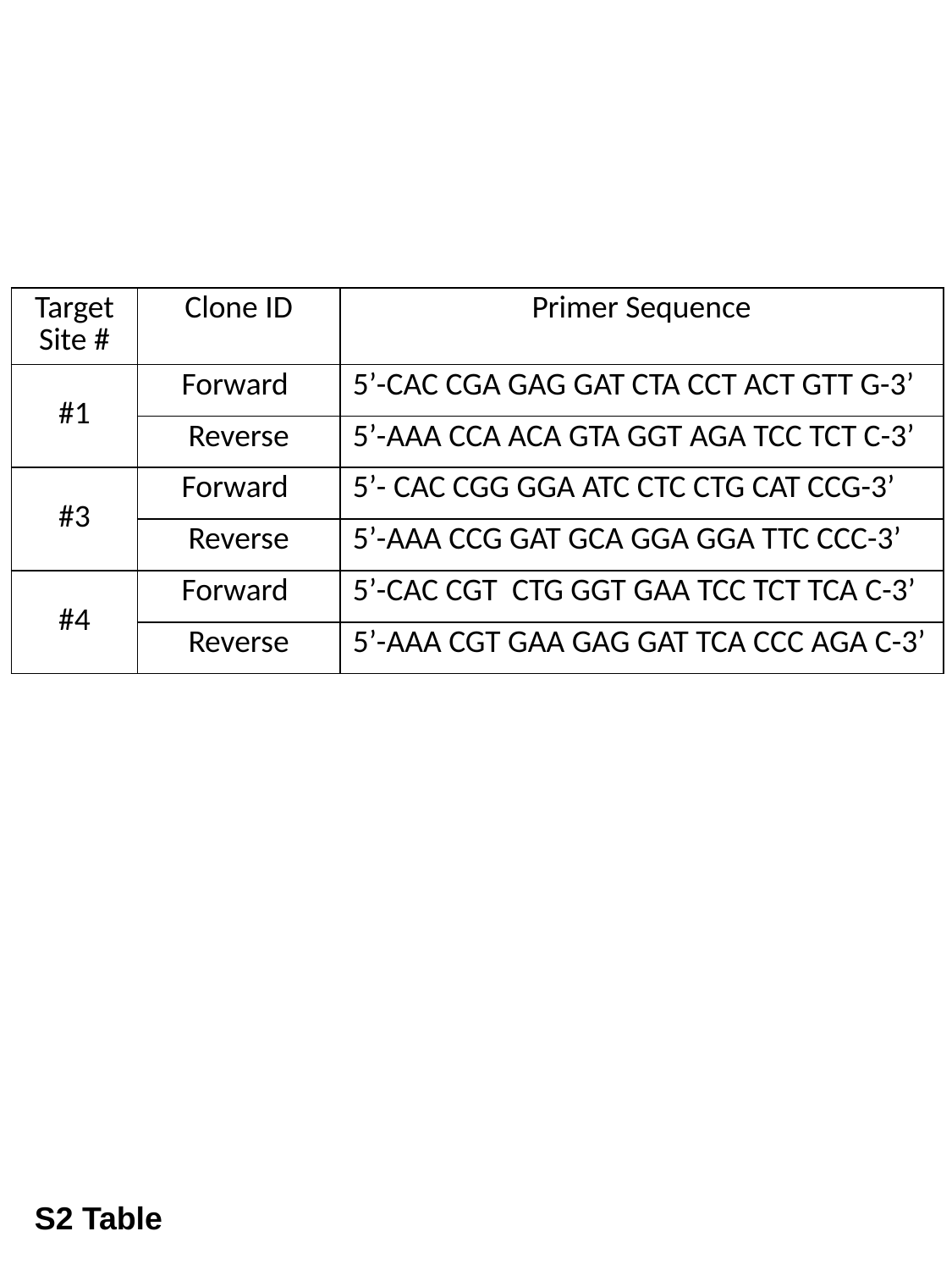

| Target Site # | Clone ID | Primer Sequence |
| --- | --- | --- |
| #1 | Forward | 5’-CAC CGA GAG GAT CTA CCT ACT GTT G-3’ |
| | Reverse | 5’-AAA CCA ACA GTA GGT AGA TCC TCT C-3’ |
| #3 | Forward | 5’- CAC CGG GGA ATC CTC CTG CAT CCG-3’ |
| | Reverse | 5’-AAA CCG GAT GCA GGA GGA TTC CCC-3’ |
| #4 | Forward | 5’-CAC CGT CTG GGT GAA TCC TCT TCA C-3’ |
| | Reverse | 5’-AAA CGT GAA GAG GAT TCA CCC AGA C-3’ |
S2 Table
