# Supplementary figures and images for: Expression of Concern: Differential expression and function of CAIX and CAXII in breast cancer: A comparison between tumorgraft models and cells
Source: PLoS One. 2025 Jul 3;20(7):e0327518. doi: 10.1371/journal.pone.0327518 (PMC12225858; doi:10.1371/journal.pone.0327518)

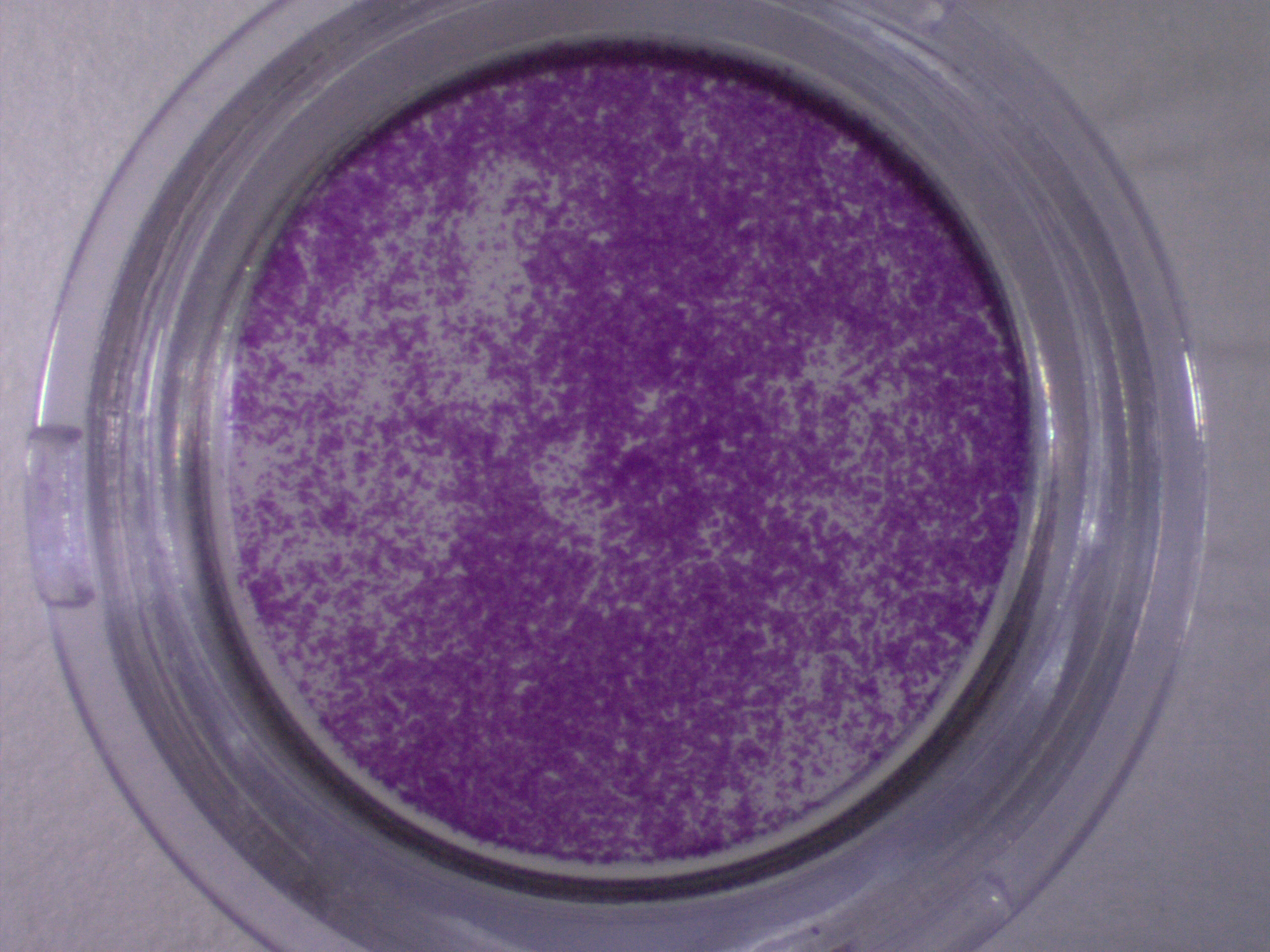

Supplement: S5 File — (TIFF) [file pone.0327518.s005.tiff]

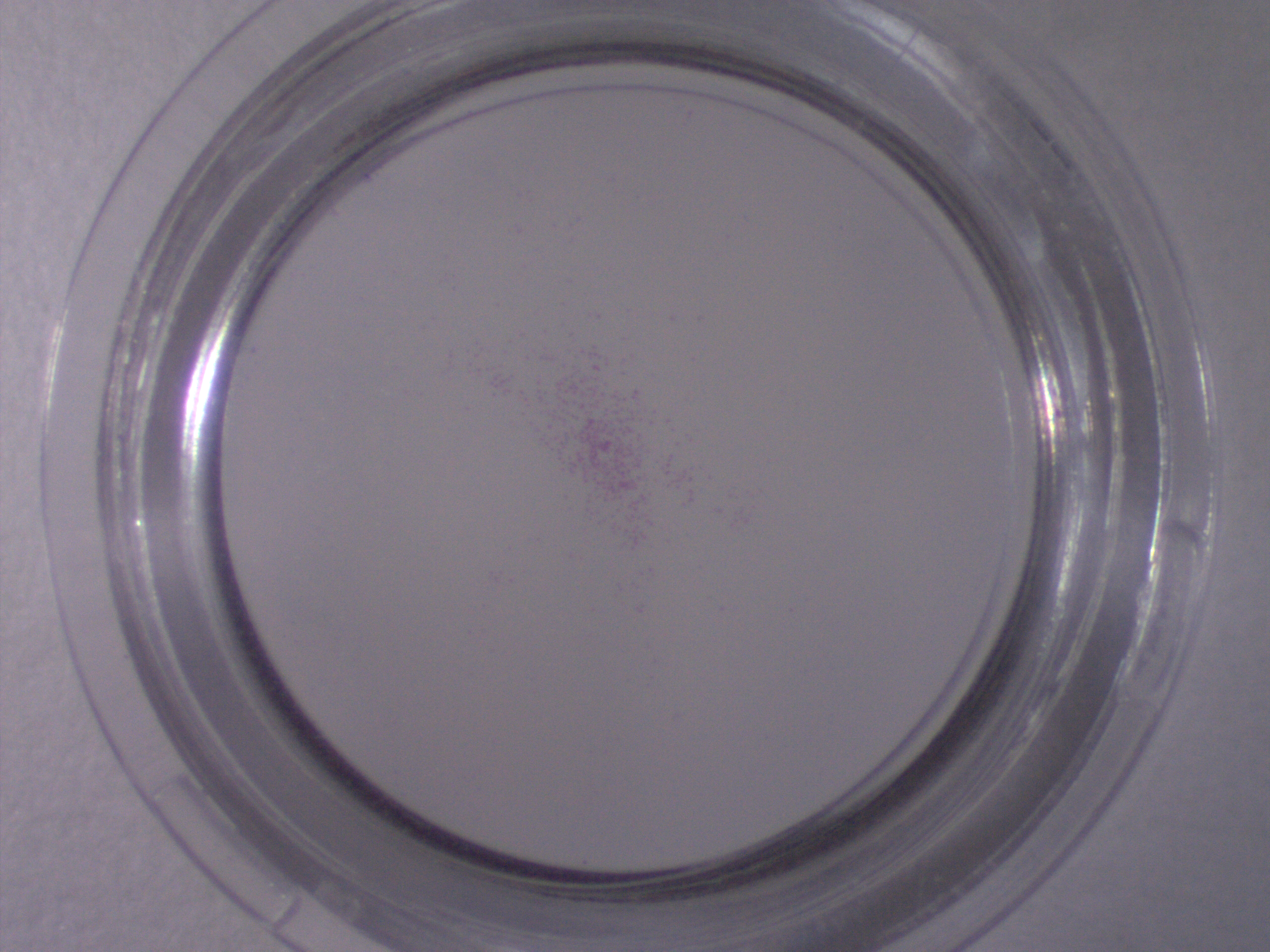

Supplement: S6 File — (TIF) [file pone.0327518.s006.tif]

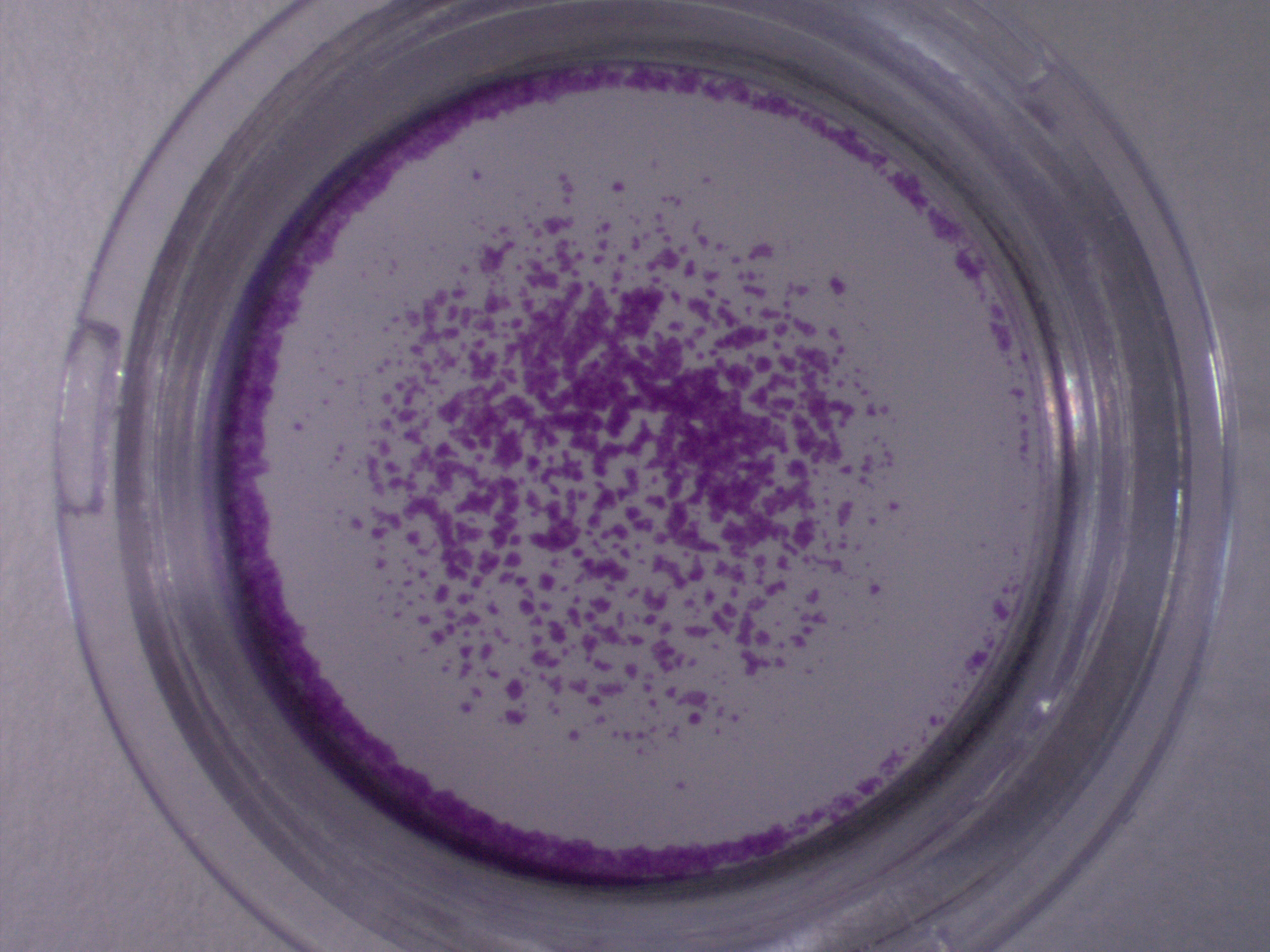

Supplement: S7 File — (TIFF) [file pone.0327518.s007.tiff]

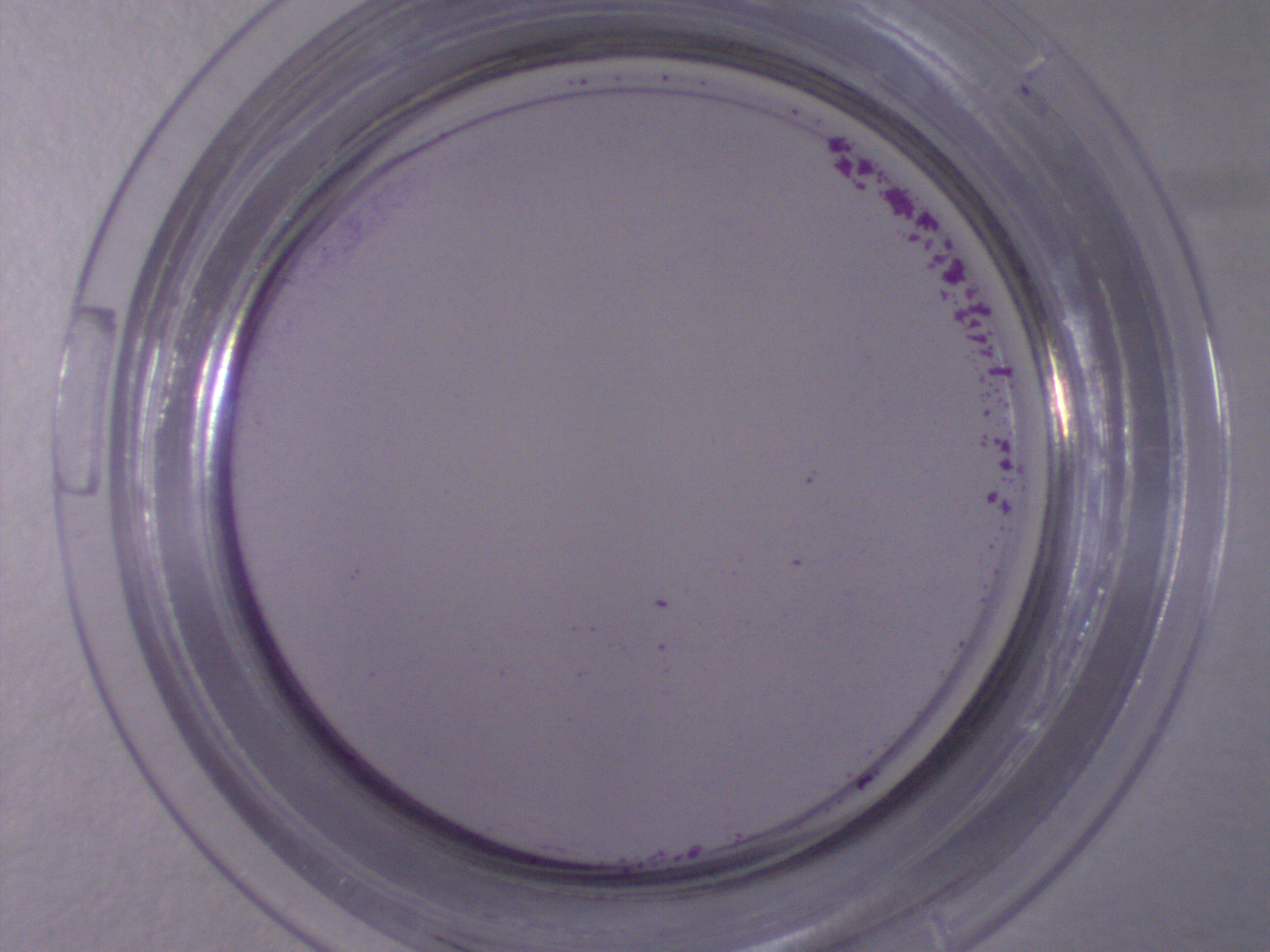

Supplement: S8 File — (TIF) [file pone.0327518.s008.tif]

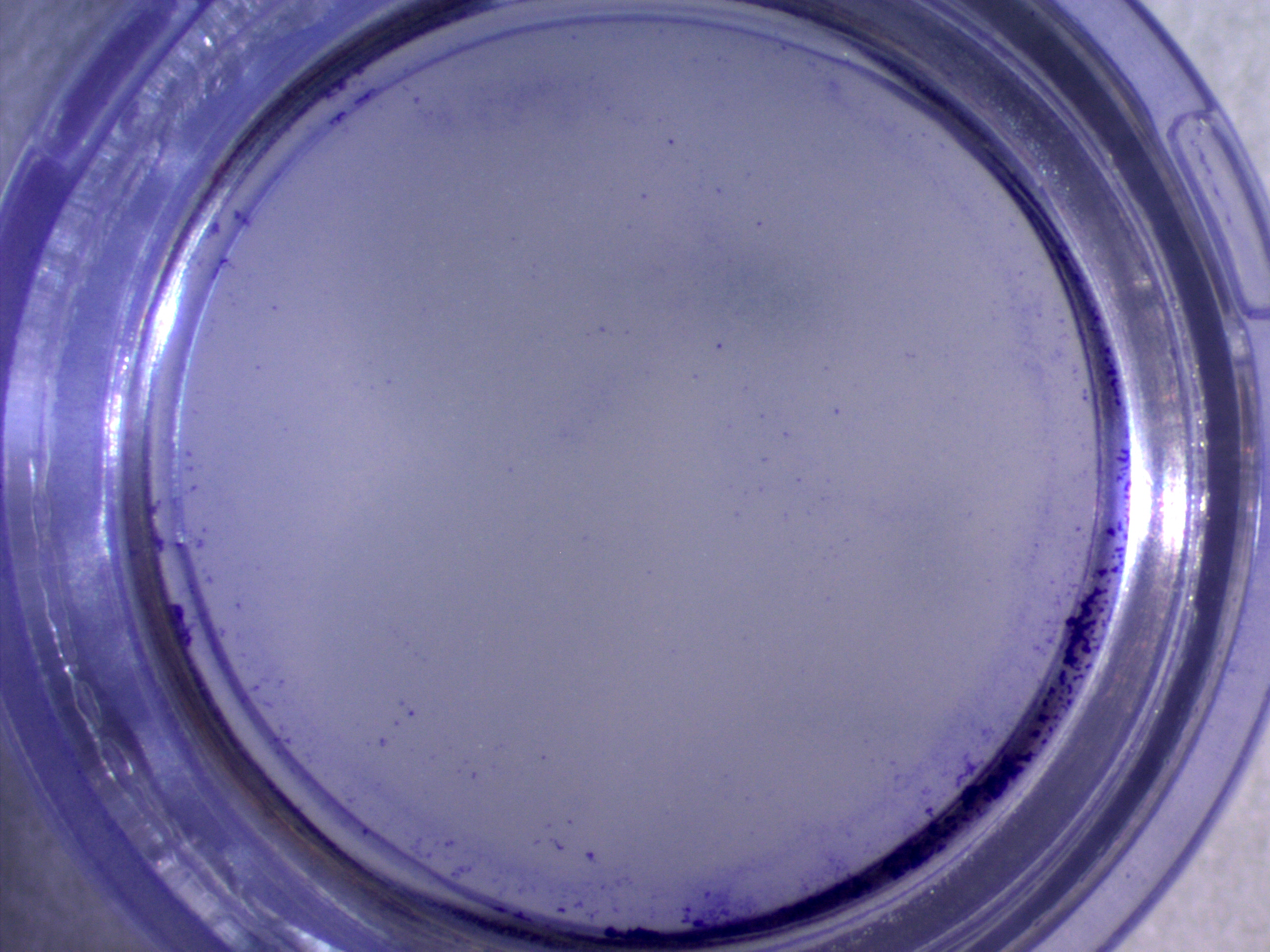

Supplement: S9 File — (JPG) [file pone.0327518.s009.jpg]

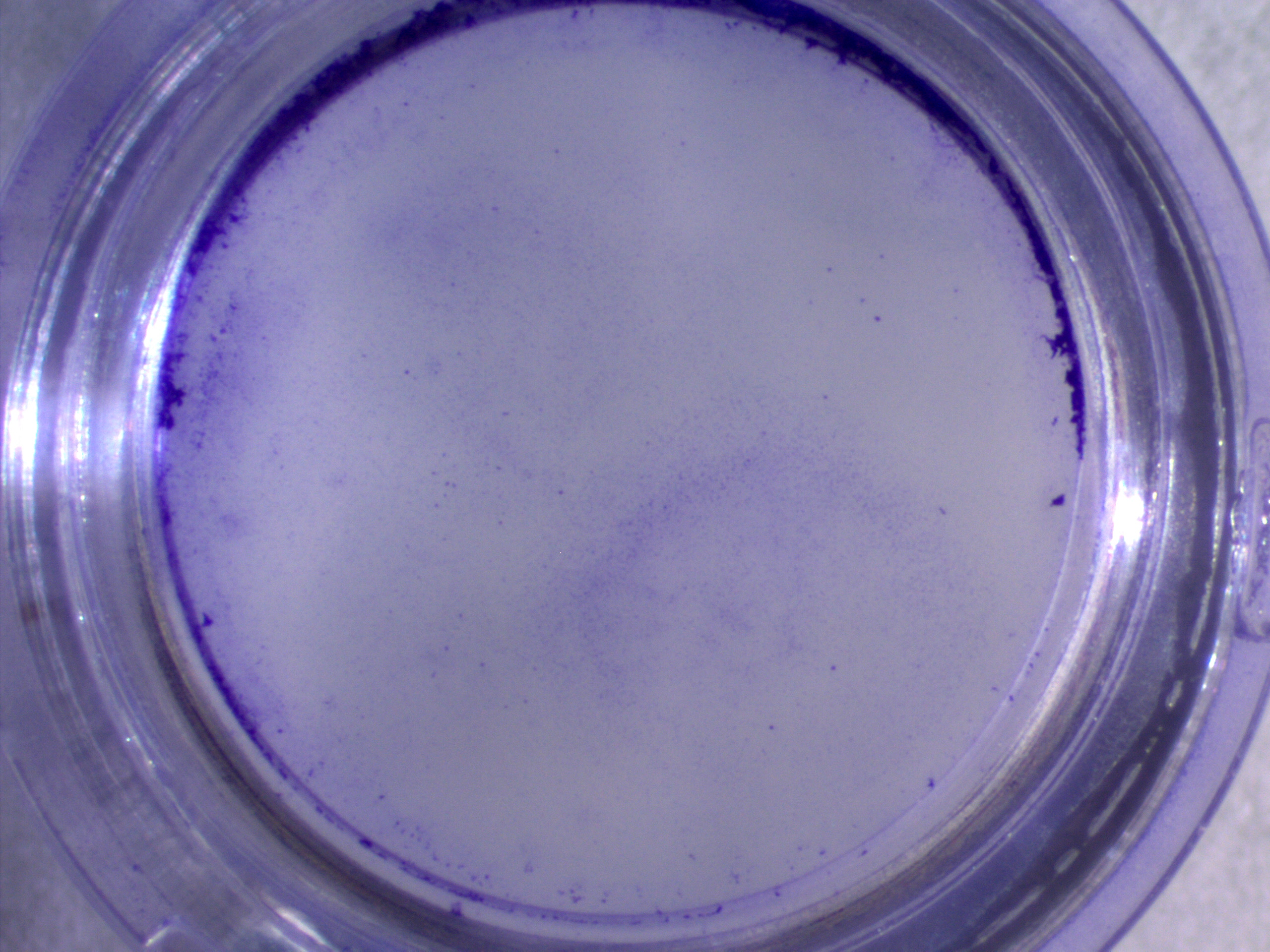

Supplement: S10 File — (JPG) [file pone.0327518.s010.jpg]

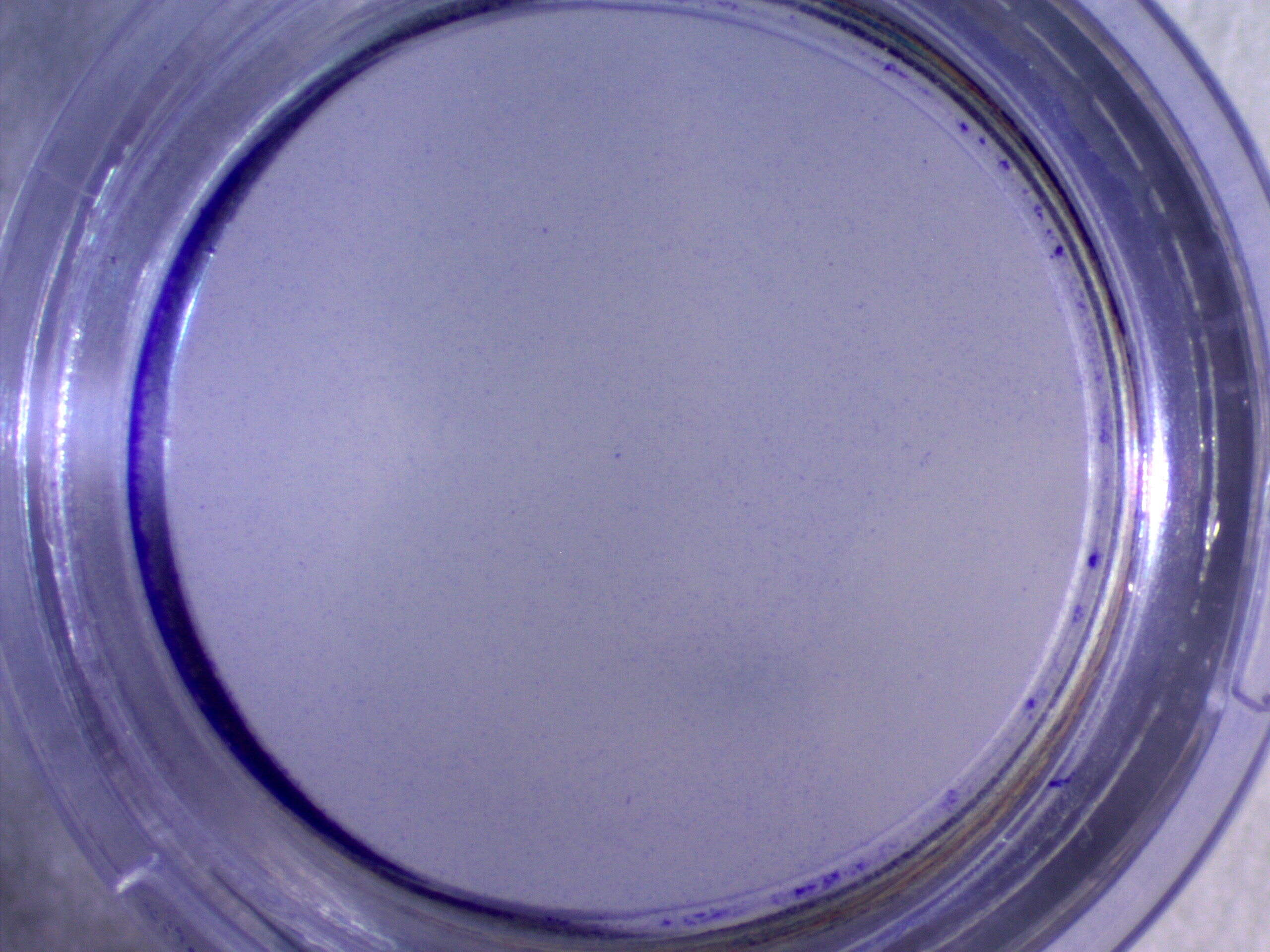

Supplement: S11 File — (JPG) [file pone.0327518.s011.jpg]

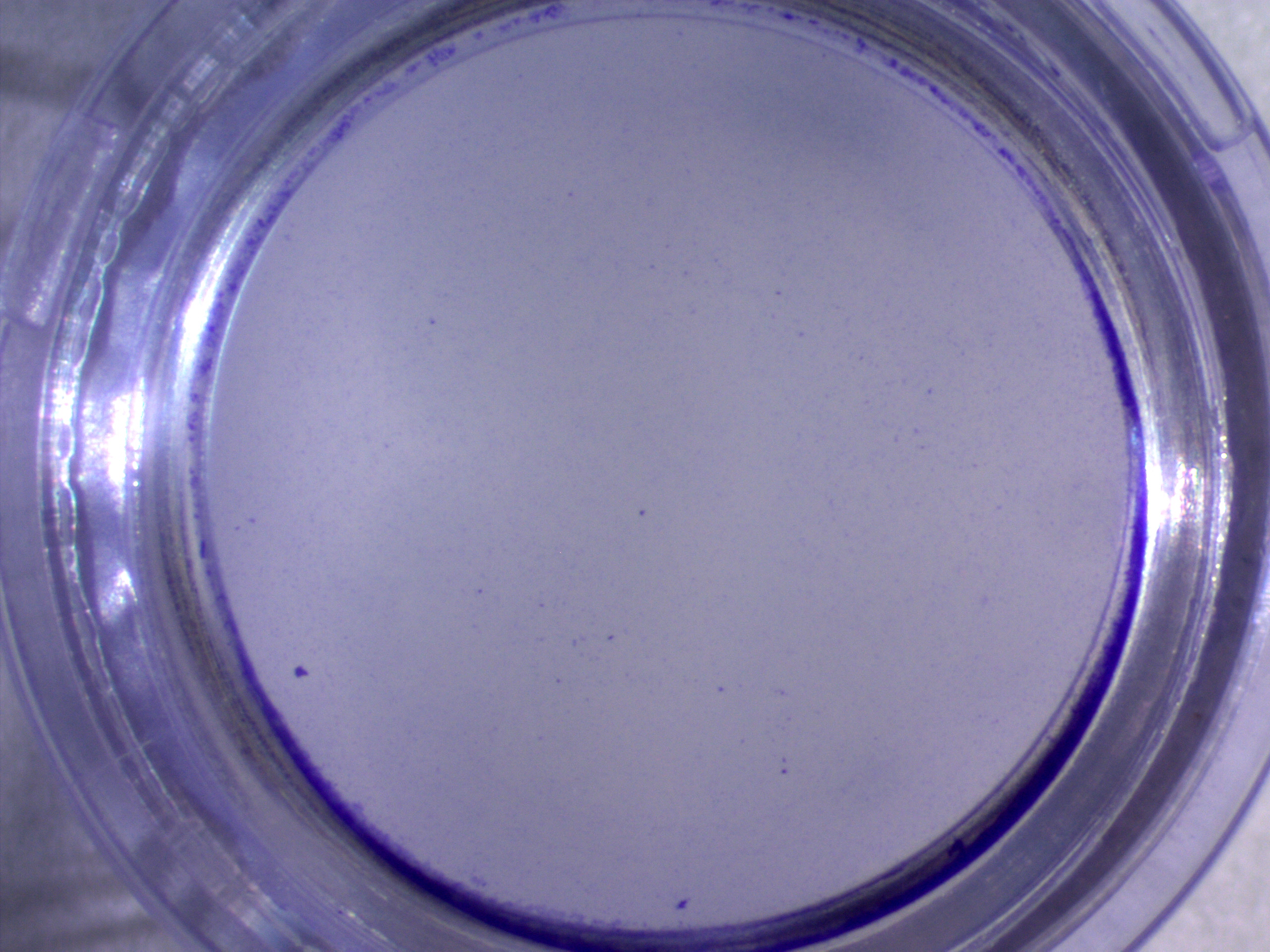

Supplement: S12 File — (JPG) [file pone.0327518.s012.jpg]
